# Beyond microroughness: novel approaches to navigate osteoblast activity on implant surfaces

**DOI:** 10.1186/s40729-024-00554-x

**Published:** 2024-07-05

**Authors:** Takanori Matsuura, Keiji Komatsu, James Cheng, Gunwoo Park, Takahiro Ogawa

**Affiliations:** 1 Weintraub Center for Reconstructive Biotechnology, UCLA School of Dentistry, 10833 Le Conte Avenue B3-087, Box951668, Los Angeles, CA 90095-1668 USA; 2 Division of Regenerative and Reconstructive Sciences, UCLA School of Dentistry, Los Angeles, USA

**Keywords:** Osseointegration, Bone and implant integration, Meso-structuring, Nanotechnology, UV photofunctionalization

## Abstract

Considering the biological activity of osteoblasts is crucial when devising new approaches to enhance the osseointegration of implant surfaces, as their behavior profoundly influences clinical outcomes. An established inverse correlation exists between osteoblast proliferation and their functional differentiation, which constrains the rapid generation of a significant amount of bone. Examining the surface morphology of implants reveals that roughened titanium surfaces facilitate rapid but thin bone formation, whereas smooth, machined surfaces promote greater volumes of bone formation albeit at a slower pace. Consequently, osteoblasts differentiate faster on roughened surfaces but at the expense of proliferation speed. Moreover, the attachment and initial spreading behavior of osteoblasts are notably compromised on microrough surfaces. This review delves into our current understanding and recent advances in nanonodular texturing, meso-scale texturing, and UV photofunctionalization as potential strategies to address the “biological dilemma” of osteoblast kinetics, aiming to improve the quality and quantity of osseointegration. We discuss how these topographical and physicochemical strategies effectively mitigate and even overcome the dichotomy of osteoblast behavior and the biological challenges posed by microrough surfaces. Indeed, surfaces modified with these strategies exhibit enhanced recruitment, attachment, spread, and proliferation of osteoblasts compared to smooth surfaces, while maintaining or amplifying the inherent advantage of cell differentiation. These technology platforms suggest promising avenues for the development of future implants.

## Introduction

There have been considerable efforts to improve bone-implant integration, or osseointegration in the fields of dental and orthopedic implants [1–6]. However, current challenges to fully optimizing clinical outcomes of dental implants include the prolonged healing time required for osseointegration [7–9], restricted indications for implant therapy in locally and/or systemically compromised bone [10–15], and the plateaued success rate at 92% [10, 16–19]. Notably, the reported percentage of bone-implant contact (BIC) remains at 45% ± 16% [9, 20], or 50–75% [4, 9, 21, 22], far below the ideal 100% [9]. Compounding this issue is the limited advancement in implant surface technology since the advent of microrough titanium surfaces three decades ago [8, 23–29], leaving an ideal surface, especially beyond microroughness, unidentified [30].

**Fig. 1:**
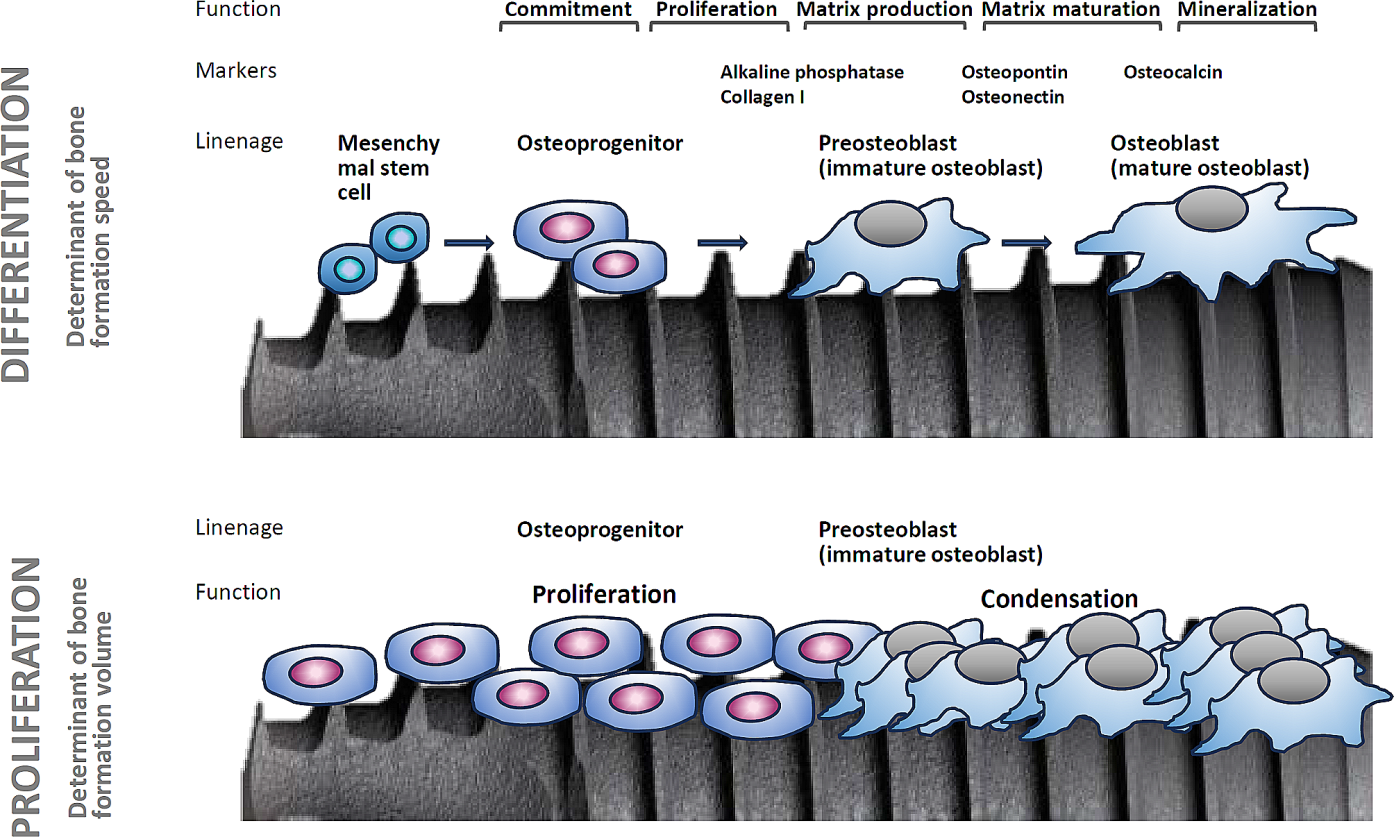
Osteoblast kinetics involve the crucial processes of proliferation and differentiation. The rate of proliferation dictates the quantity of bone formation, while the rate of differentiation determines the speed of bone formation. The property of osseointegration is influenced by how the kinetics of osteogenic cells are modulated by various factors on implant surfaces. See the main text in the Introduction for a detailed explanation

**Fig. 2:**
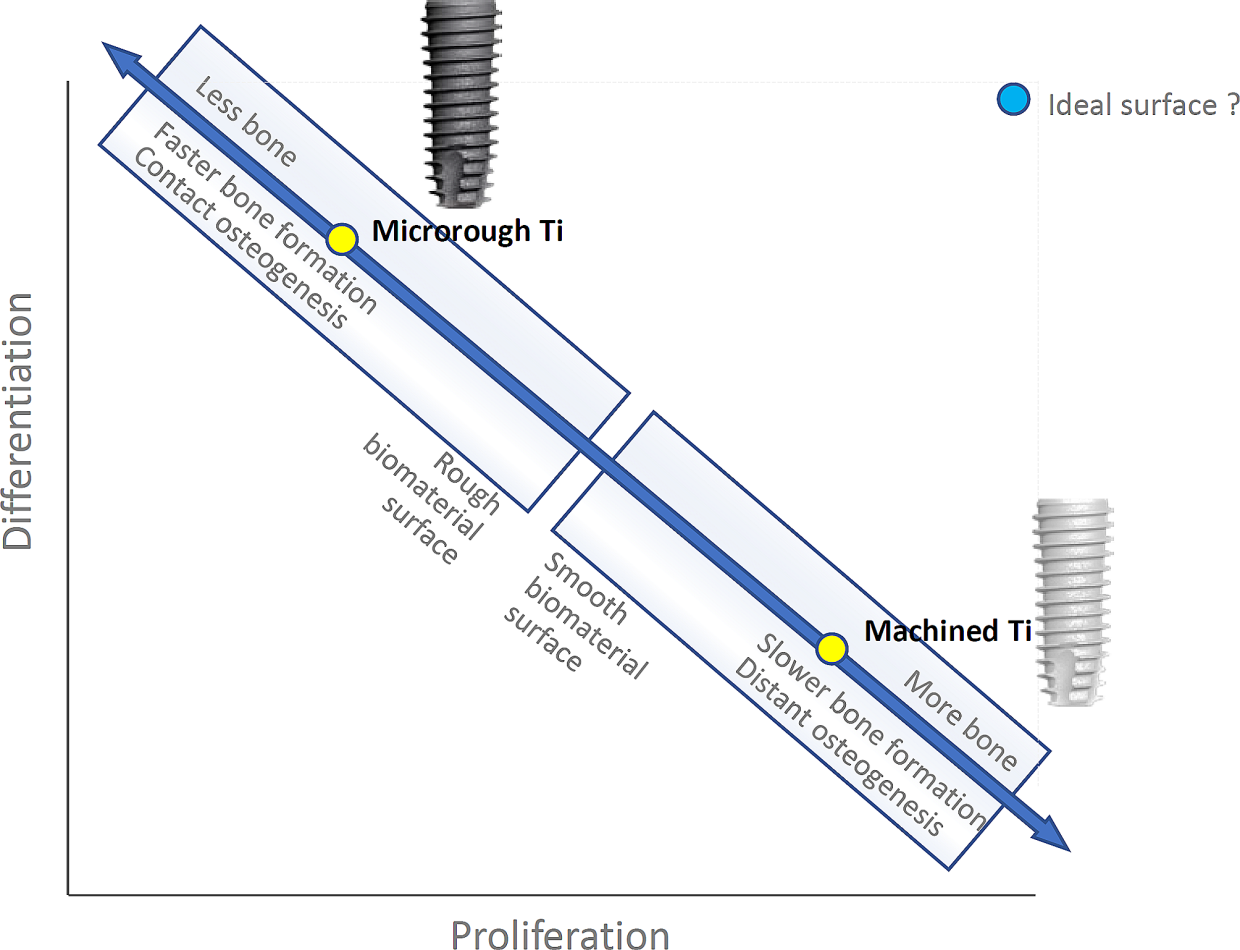
The inverse correlation in osteoblastic kinetics between the proliferation and differentiation. The proliferation is primarily responsible for the volume of bone formation, while the differentiation for the speed (See the main text in the Introduction for a detailed explanation). This inverse correlation extends analogically to the contrast between smooth and rough biomaterial surfaces, exemplified by machined titanium and microrough titanium surfaces, respectively. Ideally, titanium or biomaterial surfaces (depicted by the blue circle) should optimize both osteoblastic proliferation and differentiation to achieve rapid generation of a greater amount of bone. This paper reviews candidate strategies and scientific advancements aimed at creating such ideal surfaces

Acid etching is most commonly used to create microrough titanium surfaces [4, 8, 27, 31–38]. The process produces randomly shaped, 0.5–5 μm compartments consisting of peaks and valleys [34, 39, 40]. Despite its widespread clinical application, concerns persist regarding how surface topographies influence osteoblast kinetics, which encompass the proliferation and differentiation of osteogenic cells (Fig. 1). The rate of proliferation primarily dictates the volume of bone formation, whereas the rate of differentiation predominantly governs the speed and quality of bone formation [4, 15, 36, 41–44]. Unfortunately, there exists an inverse correlation between cellular proliferation and differentiation (Fig. 2) [45–52]. Termed the “dichotomy” of osteoblasts [42, 50], this phenomenon poses a challenge on biomaterials like titanium, creating a “biological dilemma” where osteoblasts proliferate faster but differentiate slower on smooth surfaces, resulting in delayed osseointegration [31, 42, 53–57]. Conversely, on microrough surfaces, osteoblasts proliferate slower but differentiate faster, leading to expedited but thinner bone apposition [4, 31, 36, 53, 54, 58].

The molecular pathways governing this dichotomy kinetics are relatively well understood. Runx2/Cbfa1/AML3 is a transcription factor and central regulator that activates and represses proliferation and differentiation during bone formation [59]. During early osteogenesis, signaling pathways supporting proliferation dominate but progressively diminish until eventual cessation during the post-proliferative stage and towards the differentiation stage [46, 47]. Runx2 activation triggers cell cycle exit by inhibiting the activity of S-phase cyclin complexes, leading to G1 phase arrest and the initiation of functional differentiation [60]. Indeed, proliferating cell nuclear antigen (PCNA) protein, a critical regulator of DNA synthesis and cyclin D1, whose co-expression reduces cell proliferation, were upregulated on microrough surfaces [44]. It is known that cyclin D1 can bind to PCNA and thereby inhibit DNA synthesis [61, 62]. Microrough surfaces also substantially impair osteoblast recruitment, as fewer cells initially attach to rougher titanium surfaces than to smoother titanium surfaces [42].

**Fig. 3:**
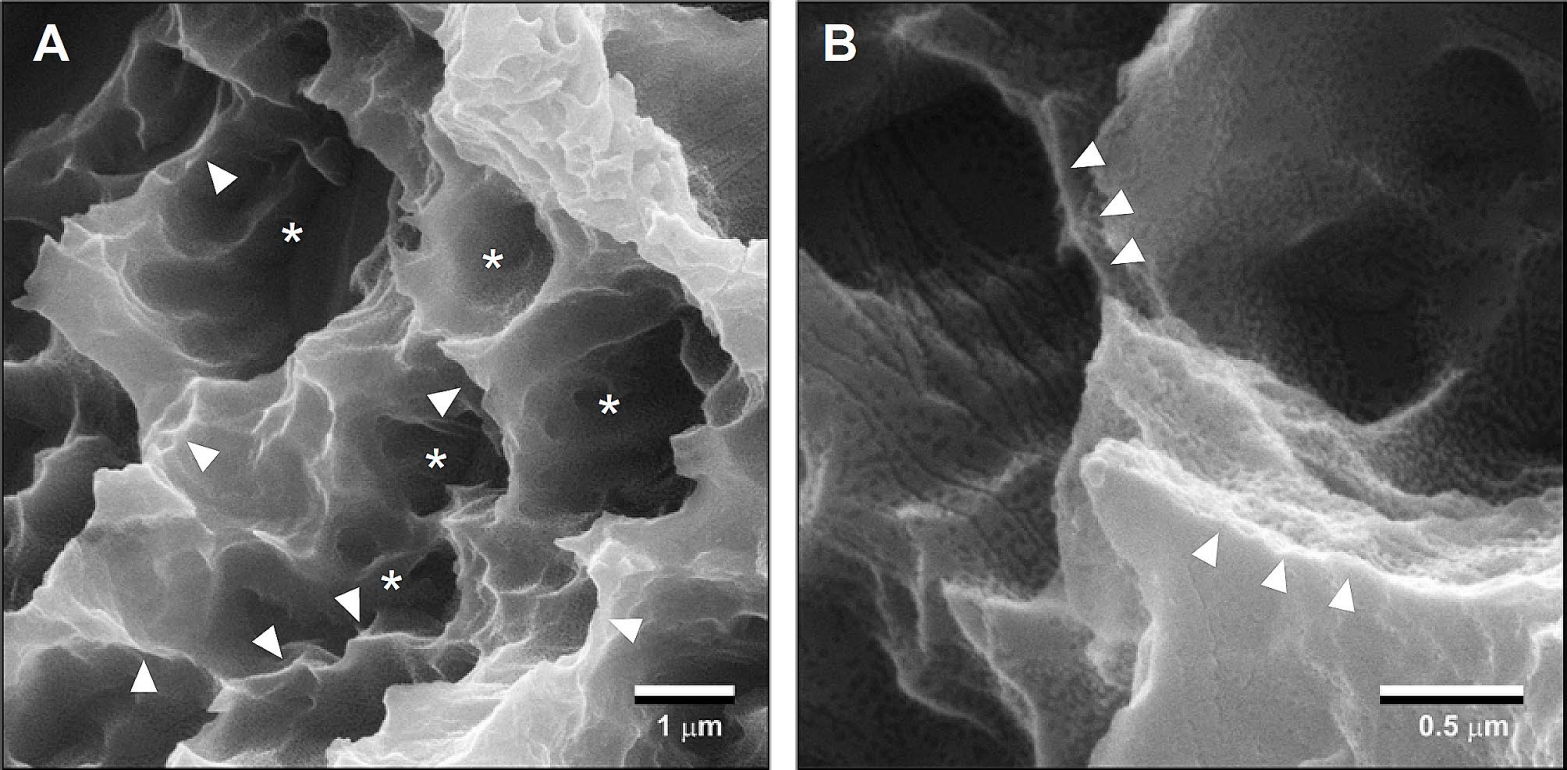
Low (**A**) and high (**B**) magnification SEM images of a typical microrough titanium surface created by sandblasting and acid etching, depicting micropits or micro-compartments made of sharp peaks and edges, referred to as a knife-edge microrough surface. Asterisks indicate micro-compartments or pits, while white arrowheads indicate knife-edges

Modern implantology prioritizes expedited peri-implant bone formation by promoting functional osteoblast maturation [6, 63]. Faster bone formation also provides contact osteogenesis around implants rather than distant osteogenesis [64–67], minimizing soft tissue intervention between the implant surface and bone (Fig. 3). Intrinsic mechanical properties of bone, such as the harness, elastic modulus, and adhesion strength to materials are also associated with the speed of osteoblastic differentiation and bone formation [39, 68–70]. These events are not mutually exclusive, and microroughened surfaces can successfully promote these outcomes [36, 39, 71–74]. Further, roughened surfaces increase the load-bearing capacity of implants, as the surfaces provide increased mechanical interlocking between implant surface and bone and even between implant surface and cells [75, 76]. Rough surfaces also promote cellular mechano-transduction to favor osteoblastic differentiation [77]. However, enhanced differentiation of osteogenic cells accompanies the drawback of reducing bone volume due to osteoblast proliferation deactivation [4, 36, 44].

Improving osseointegration involves overcoming the dichotomy kinetics of osteoblasts on roughened titanium surfaces, preserving differentiation while minimizing its impact on proliferation. Despite extensive research on factors promoting osteoblast differentiation [2, 44, 78–87], little attention has been given to promoting osteoblastic proliferation. This review explores recent advancements in nanonodular and meso-texturing, and UV photofunctionalization as potential strategies to address this challenge, offering promising avenues for rapid and increased bone generation around implants.

## Nanonodular texturing to temper microrough surfaces

### Surface morphology and biological impact

Significant attention has been devoted to the surface morphology, texture, and topography of titanium to enhance the bioactivity and osteoconductivity of implants. Starting with original machined surfaces and progressing to supra-micro-scale rough surfaces, such as titanium plasma sprayed (TPS) surfaces [88] and hydroxyapatite (HA)-coated surfaces [89], microrough surfaces have become the norm in modern implant dentistry [1, 83, 90]. Among the advancements from microrough titanium surfaces is nanonodular texturing, aimed at tempering or rounding these surfaces.

Nanonodular texturing of titanium describes the controlled creation of titanium nanonodules on microrough titanium surfaces through molecular self-assembly during TiO_2_ sputter deposition [91–93]. This technique effectively arounds and tempers the sharp peaks and edges of microrough titanium surfaces while preserving the main micro-pit configuration. Despite the increased surface roughness resulting from nanotexturing, osteoblast attachment and proliferation still significantly increase [91, 93], but not at the expense of impaired differentiation.

**Fig. 4:**
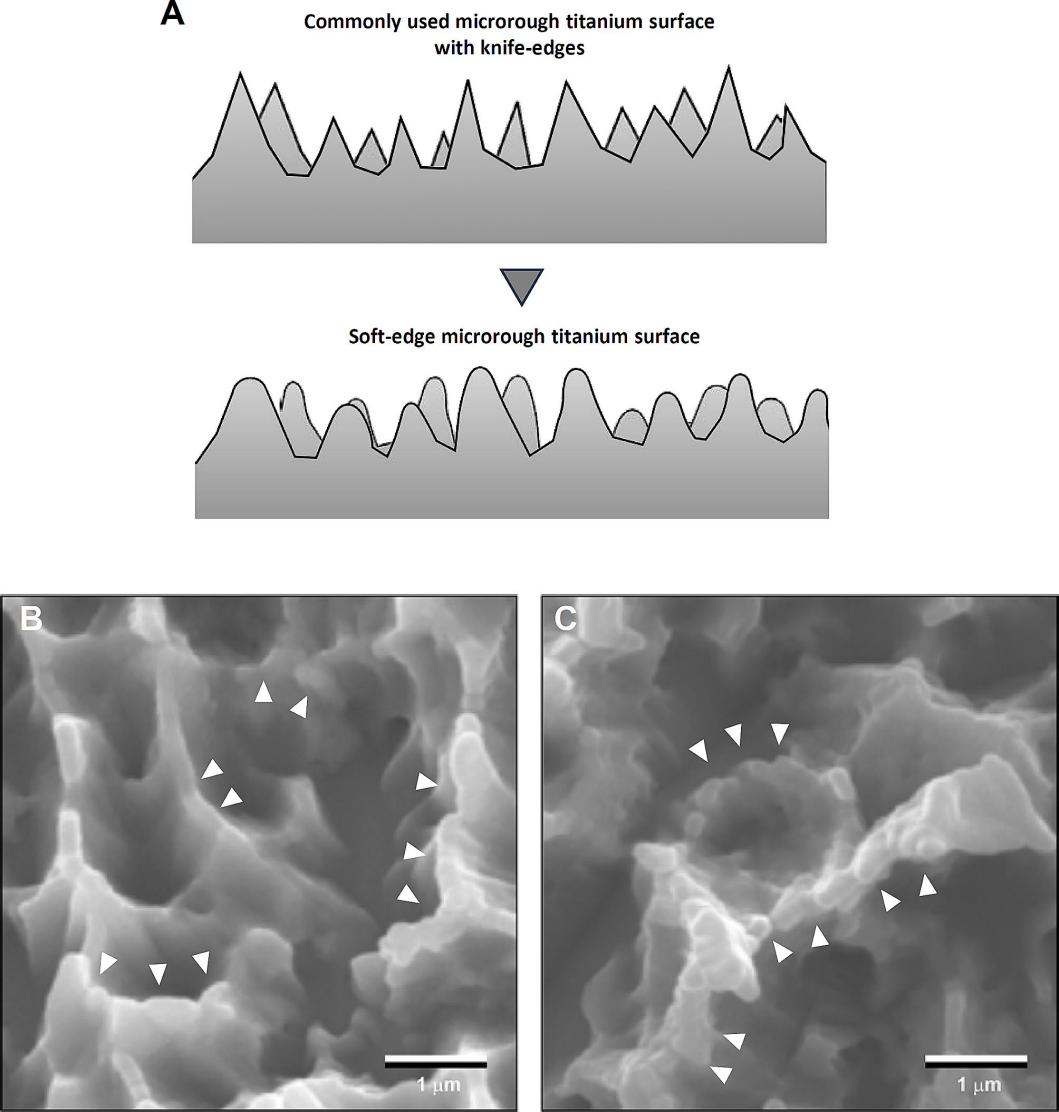
Micro-tempering as a strategy to mitigate the drawback of microrough surfaces. (**A**) An illustration of the strategy to convert knife-edges to soft-edges on microrough titanium surfaces. (**B**, **C**) SEM images of a tempered microrough titanium surface. The microrough titanium surfaces, as shown in Fig. 3, were sputter-depositioned with TiO2, resulting in the rounded peaks and edges of the original microroughness (white arrowheads) and the formation of nanonodules within the pits and along the peaks and flanks, establishing the soft-edge micro-nano hybrid surface. Note the main configuration of the original micropits is preserved

In the absence of nanonodular texturing, microroughened titanium surfaces exhibit sharp peaks and valleys measuring 0.5–5 μm (Fig. 3), forming micro-pits or micro-compartments with knife-edges. As illustrated in Fig. 4A, to round this sharp morphology, TiO_2_ is sputter deposited onto acid-etched, microrough titanium surfaces. This process smooths the peaks and the bottom of the pits by creating nano-nodules while simultaneously preserving existing micro-pits outlines, thereby establishing a micro-nano hybrid topography with soft-edges as confirmed by scanning electron microscopy (SEM) imaging (Fig. 4B, C). Quantitative roughness analysis reveals that nanonodular texturing increases the average roughness (Sa) and the peak-to-valley roughness (Sz) by 2–4 times compared to microrough surfaces [50, 91]. The titanium nanonodular structures resemble calcium crystalline accretions found in the mineralized matrix formed by cultured osteoblasts [50, 91], presenting a biomimetic topography favorable for osteoblastic activity.

In vitro studies have demonstrated that three to five times more osteoblasts attach to the soft-edge hybrid surfaces during the initial stages of culture (6 h) than to original microrough surfaces with knife-edges, despite the significantly increased roughness [50, 91]. The effect persists since, after 24 h, hybrid surfaces still harbored twice the number of adherent cells. Furthermore, in these experiments, there were double the number of proliferated cells during subsequent culture (days 2 and 6) on soft-edge hybrid surfaces [50, 91]. Notably, the cell density on soft-edge hybrid surfaces matches that on machined, smooth surfaces [50]. Thus, the tempered morphology of microrough surfaces through nanonodular texturing overcomes the inverse kinetics of osteoblasts and completely mitigates the impaired attachment and proliferation of osteoblasts on microrough surfaces. Despite the increase in cell proliferation, functional differentiation was promoted on the soft-edge hybrid surface, as evidenced by upregulation of osteogenic extracellular matrix genes and calcium deposition, indicating that the soft-edging resolves the biological dilemma of microrough surfaces [50, 91]. Additionally, in an in vivo rat femur model, the biomechanical strength of osseointegration is three-fold greater for soft-edge hybrid implant surfaces than knife-edge microrough implant surfaces [91].

### Cell behavior supporting increased attachment and proliferation

In general, roughened surfaces impede cell spreading compared to smooth surfaces. Experimental comparisons between microrough and machined smooth surfaces revealed that cellular areas and perimeters were 50–70% smaller on microrough surfaces than on smooth surfaces after an initial six-hour culture [42]. In contrast, hybrid implant surfaces with soft edges induced cellular behaviors that enhanced osteoblastic attachment, adhesion, and proliferation compared to microrough surfaces. Cells cultured on soft-edge surfaces exhibited significantly larger sizes than those on microrough and machined surfaces, showcasing robust cytoskeletal and cytoplasmic projections [91, 94]. Moreover, cells on soft-edge hybrid surfaces developed actin-based structures, including filopodia and lamellipodia, indicative of active migration and proliferation. The extensive expression of vinculin, a focal adhesion protein [95, 96], suggested that cells behaved as if they were interacting with both cells and the extracellular matrix before clustering and colonization [50], potentially further enhancing cellular attachment, adhesion, and proliferation [91, 97]. Vinculin indeed responds to nanofeatured surfaces [98]. Increased protein adsorption by 2-2.5 times on the soft-edge microrough surfaces compared to knife-edge microrough surfaces may have also contributed to the manifestation of these enhanced cell behaviors [91].

### Tempered hybrid configuration optimization

In optimization studies, the size of nanonodules ranged from 100 nm to 500 nm, achieved by controlling the sputter deposition time with a fixed deposition rate was 18.5 Å/minute [91]. The estimated deposition time and the size of nanonodular formation were nearly perfectly correlated (*R* = 0.99), providing the technology validation. The degree of surface roughness measured by the average roughness (Sa) and the peak-to-valley roughness (Sz) did not necessarily correlate with the nodule size (Sz) and was highest when the nodules were 300 nm [91]. Moreover, cellular attachment and proliferation were highest on the 300 nm hybrid surface, contradicting the trend where rougher surfaces typically reduce cellular attachment and proliferation [91]. Osteoblastic gene expression was also most upregulated on these surfaces. Thus, osteoblastic behavior and growth exhibit unexpected patterns on tempered hybrid surfaces, resulting in biological enhancement and increased roughness, along with the formation of undercuts on the titanium surfaces, consequently leading to significantly improved osseointegration in vivo [91]. Interestingly, the tempered microrough titanium surface has been found to decrease proliferation and function in fibroblasts, offering an additional advantage for enhanced osseointegration without soft tissue invasion [50].

### Versatility of the nanotechnology-driven tempering strategy

The strategy of mitigating the inverse correlation between osteoblast proliferation and differentiation has been explored on various surfaces. Titanium treated with sandblasting and hydrofluoric acid also benefits from nano-nodular texturing, providing soft-edges and nanostructures [92]. Furthermore, other biomaterials, including CoCr alloy, NiCr, ceramic (SiO_2_) surfaces, and organic and polymeric surfaces such as polystyrene cell culture dishes, poly-lactic acid (PLA), and collagen membranes, can be nano-textured with rounded edges, indicating the generalizability of the protocol across different materials [92].

### Unique biological impacts of nanonodule-tempered surfaces

Not all nano-texturing techniques effectively mitigate the adverse effects of microrough titanium surfaces. For instance, submerging microrough titanium surfaces in an isotonic NaCl solution produces a surface characterized by micro-pits typical of acid-etching and nano-deposits induced by NaCl crystallization [99, 100]. However, the number of osteoblast-like osteosarcoma MG63 cells attaching to this nano-featured surface 24 h after seeding was 30–50% lower than those attaching to microrough implants [54, 101], despite an increase in differentiation markers [101–103].

Hydrofluoric acid treatment of sandblasted titanium forms micro-nano-hybrid rough surfaces, with randomly-shaped nano-textures on the microroughness [21, 85, 104]. After 48 and 72 h of culturing, cells seeded on surfaces created by sandblasting alone with 75 μm particles exhibited the highest proliferation rates, while hybrid surfaces led to decreased cell proliferation as fluorine concentration increased, by up to approximately 30% [85], although the differentiation was promoted [86]. Thus, not all nano-features can overcome the biological dilemma of osteoblasts, underscoring the unique efficacy of tempered microrough configurations with soft edges.

### Another explanation of the benefit of tempered microrough surfaces

Improved chemistry may also contribute to the increased osteoblast attachment and proliferation observed on tempered microrough titanium surfaces. Surface oxygen plays a crucial role in promoting osteoblast proliferation on implant surfaces, facilitating more pronounced cellular spreading and phenotypic changes [105–107]. A study compared osteoblastic attachment and growth on machined titanium specimens sputter-coated with Ti and TiO2, with the TiO2-coated surface having four times greater surface oxygen [37]. Cell proliferation was faster on TiO2 surfaces up to 60%, while the differentiation was not significantly modulated. Additionally, advancements in technology, such as pico-technology, enable the deposition of an extremely thin layer of molten TiO_2_ (300 picometers to 6.3 nanometers) onto microrough titanium surfaces without altering their topography [108]. On these surfaces, the number of osteoblasts attaching after 6 h of culture increased linearly with the surface oxygen percentage. Furthermore, robust cytoplasmic projections were observed spreading from the osteoblast bodies. Thus, the increased surface oxygen may partially explain the advantages of the TiO_2_ nanonodule-mediated microrough tempering.

## Meso-structuring to increase the surface area

### Surface morphology and biological impact

Surface morphology of biomaterials encompasses various features such as topography, pattern/order, roughness, spacing, and even mechanical properties. Meso-structuring of titanium, as discussed here, aims to optimize both topography and spacing factors to attract, settle, and cluster cells effectively.

**Fig. 5:**
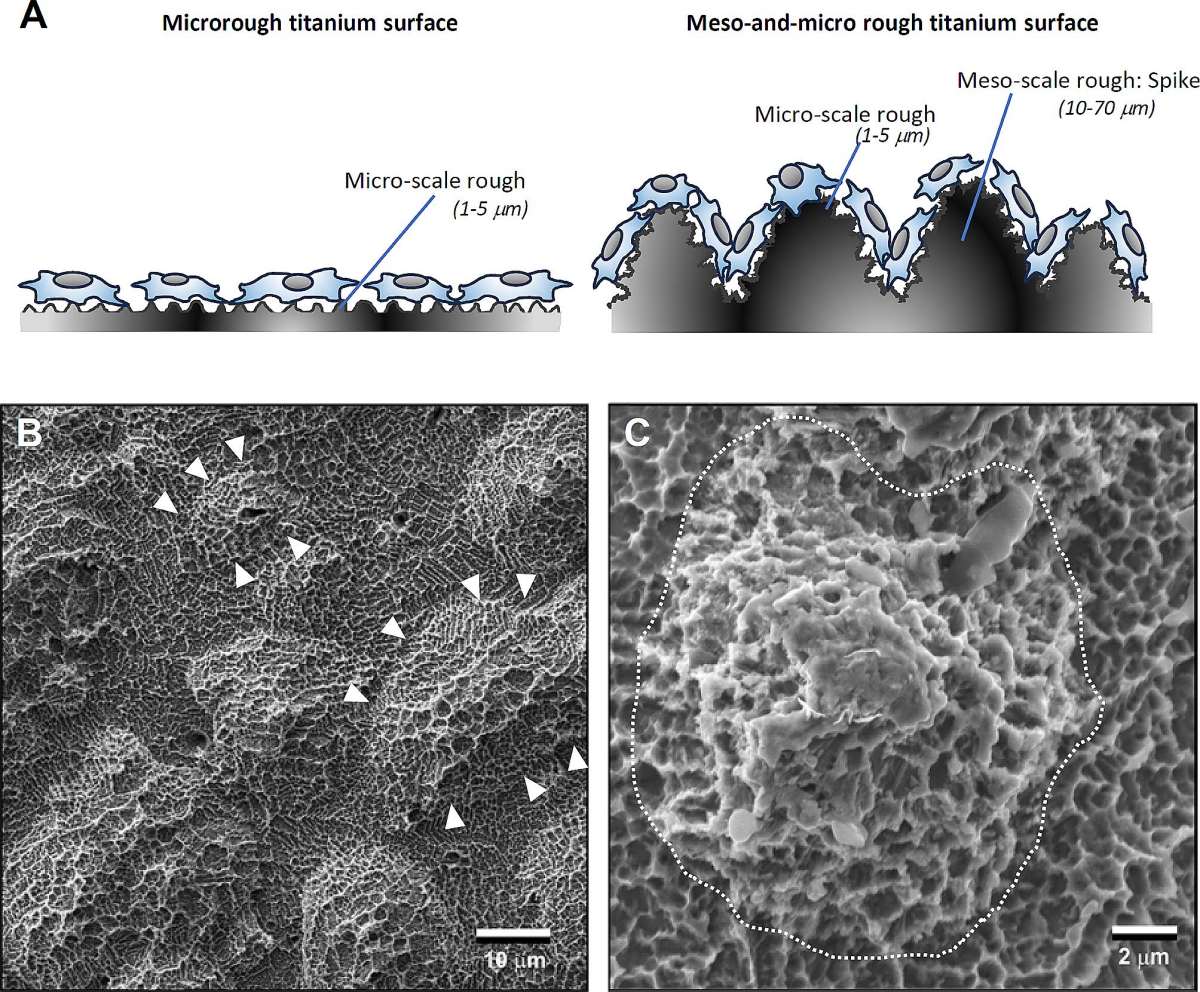
Meso-structuring as a strategy to mitigate the drawbacks of microrough surfaces by increasing the surface area. (**A**) A schematic of the meso-textured titanium surface to provide more surface for cells to attach, settle, and proliferate. (**B**) A low SEM image of a meso-structured titanium surface. High-temperature acid etching created meso-scale spikes (areas surrounded by white arrowheads) all over the surface, along with micropits similar to the typical microrough titanium surface shown in Fig. 3. (**C**) A focused SEM image of a meso-spike (area surrounded by a dotted line). Nano-scale structures in polymorphic forms are visible within the micropits, creating a meso-, micro-, and nano-hierarchical rough titanium surface

As illustrated in Fig. 5A, considering that osteoblasts typically range from 30 to 80 μm in diameter, increasing the surface area at the meso-scale (> 10 μm) rather than at the micro- or nano-scale may prove to be an effective strategy for optimal cell settlement and colonization on implant surfaces. Although there are limited studies on meso-surface topographical alterations [109], one study achieved meso-texturing of titanium through aggressive, high-temperature acid etching, resulting in the creation of meso-scale (> 10 μm) spikes alongside typical micro-pits on titanium surfaces, thereby generating a three-layered (meso-, micro-, nano-) rough titanium surface [40]. The meso-roughness was characterized by vigorous protrusions of 10–70 μm in width and height, while the micro-roughness resembled the sharp-edged micro-pits formed by acid-etching, and the nano-roughness was represented by polymorphic structures (see Fig. 5B, C). Compared to regular acid-etched, microrough surfaces, the average roughness increased up to tenfold. Since the surface was created via a subtractive method like acid-etching, there are no concerns regarding structure delamination or dissociation, as reported in titanium surfaces created by additive methods such as titanium plasma spray or hydroxyapatite coating.

Despite the substantial increase in roughness, the number of primary osteoblasts attaching over 24 h was not compromised, and their cellular behavior significantly improved on meso-textured surfaces [40]. Furthermore, the surface promoted functional differentiation, indicating significant mitigation of the limitations of microrough surfaces while simultaneously enhancing their advantages [40]. Due to the remarkable increase in surface area, more osteoblasts attach and proliferate on meso-structured surfaces compared to microrough surfaces as strategized in Fig. 5A, thereby offsetting the drawbacks associated with rougher surfaces.

### Meso-structuring and roughness tempering on zirconia

A similar approach to increase surface area and temper roughness configuration has been applied to zirconia surfaces, which are increasingly utilized in the dental implant market due to their advantageous properties, including a color similar to bone and teeth, reduced allergic reactions compared to titanium, and reportedly better biocompatibility with gingival tissues [110–113].

**Fig. 6:**
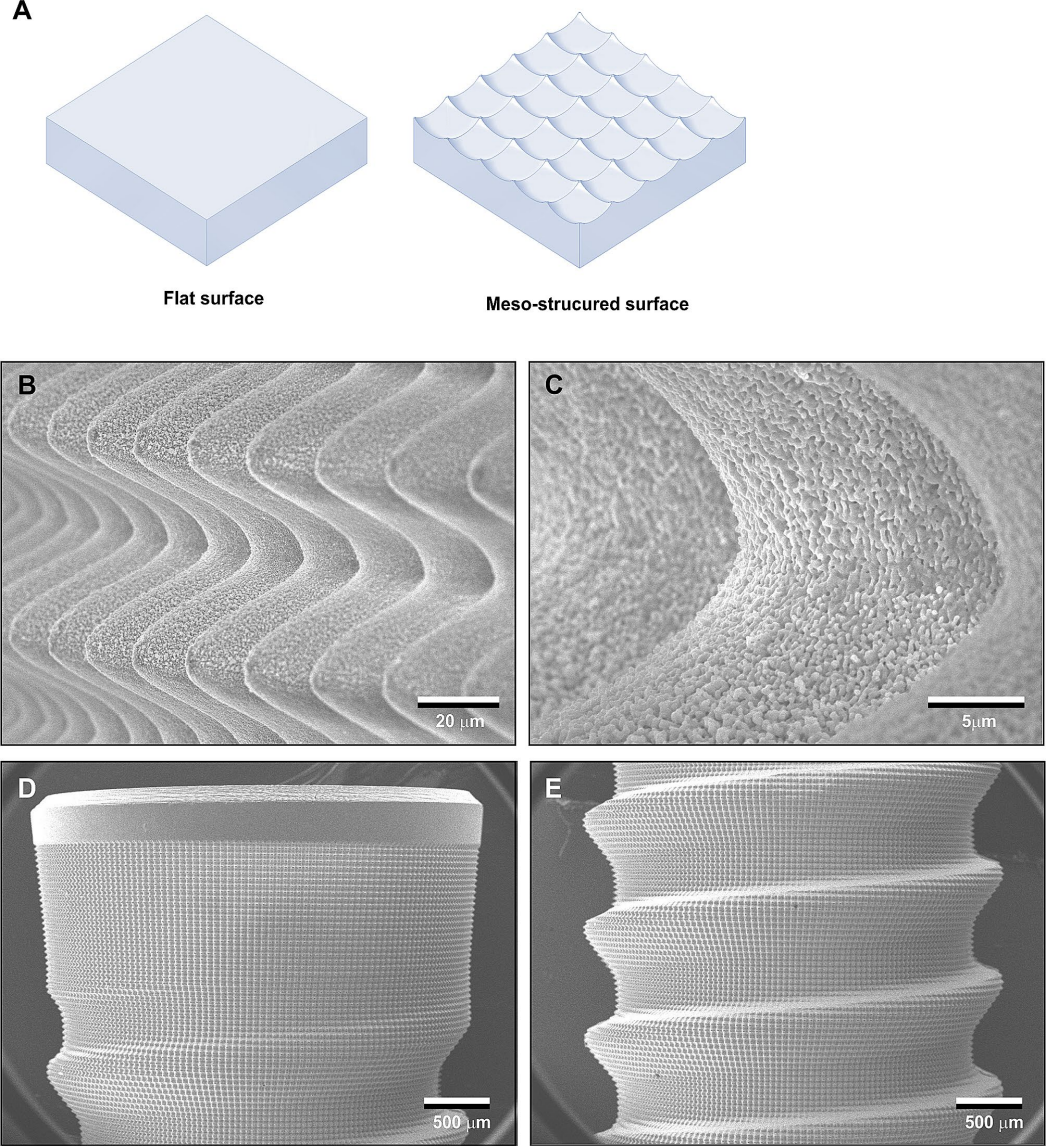
Meso-structuring on zirconia. (**A**) Strategy to create meso-scale spikes via crisscrossing, solid-state laser etching. (**B**, **C**) SEM images of the laser-created meso-spikes on zirconia with simultaneously created nanonodules. The spikes and nodules mimic cactus and trabecular bone, respectively, creating a dual bio-inspired zirconia surface. (**D**, **E**) The cactus-inspired meso-structing successfully accomplished on prototype, zirconia dental implants

To introduce meso-scale grooves, injection-molded yttria-stabilized tetragonal zirconia polycrystal (Y-TZP) was etched using a solid-stage laser [55–57]. Additionally, crisscrossing laser-etching was employed to create meso-scale peaks (Fig. 6A). The resulting etched surface displayed an array of 50–60 μm wide, variably high cactus-inspired spikes (Fig. 6B, C). These meso-spikes featured 200–300 nm trabecular bone-inspired interwoven nodular structures throughout the surface (Fig. 6C). All surface configurations were rounded and curved to minimize sharp edges and angles, akin to the nano-nodular structuring protocol used on titanium. The height and width of meso-spikes were meticulously controlled.

Compared to polished smooth zirconia surfaces, the meso-spikes significantly increased surface roughness, with the average roughness of the 40 μm-high spike surface rising from 0.10 to 7.7 μm, and the surface area increasing 2.5-fold [57]. Interestingly, despite the increase in surface roughness with the elevation of meso-spikes, the number of osteoblasts attaching at 24 h remained uncompromised [57]. Thus, the benefits of the increased surface area outweighed the negative impact of the increased surface roughness. As expected, the rate of osteoblast differentiation surged on the meso-and-nano hybrid surface, leading to elevated alkaline phosphate production and calcium deposition in vitro, as well as enhanced osseointegration in vivo [57].

Solid-state laser etching has demonstrated feasibility in creating prototype dental implants with the bio-inspired meso- and nano-hybrid surface morphology (Fig. 6D, E). A recent computational fluid dynamic (CFD) modeling study unveiled that this specific meso-structuring enhanced blood and protein recruitment and retention at the implant interface by altering and slowing down the vector of blood flow [55]. Consequently, osseointegration around meso- and nano-structured zirconia implants was notably augmented [57].

The surface features and properties of titanium and zirconia presented in this paper, along with their representative effects on osteogenic cells, are summarized in Table 1. Surface technology has evolved such that advanced surfaces mitigate or overcome the biological disadvantages of their predecessors.

**Table 1:** Surface features and properties of titanium and zirconia introduced in this review and their biological effects on osteogenic cells

Surface/ material	Machined Ti	Microrough Ti	Soft-edge microrough Ti	Meso-and-micro rough Ti	Machined Zr	Meso-and-nano Zr	UV-photofunctionalized Ti
Technique	Machine-milling	Acid-etching, sandblasting, etc.	Ti sputter-coating on microrough Ti	High-temperature acid-etching	Machine-milling	Laser-etching	Ultraviolent light treatment
Average roughness (Sa)	0.1–0.2 μm	1.0–2.0 μm	2.0–4.0 μm	5.0–7.0 μm	0.1–0.2 μm	7.0–10.0 μm	No change
Surface physicochemistry	TiO2	TiO2	Deposited TiO2	TiO2	YTZP	YTZP	TiO2 with reduced carbon. Superhydrophilic
Osteogenic cell activity	Attachment + Proliferation + Differentiation −	Attachment − Proliferation − Differentiation +	Attachment + Proliferation + Differentiation +	Attachment + Proliferation + Differentiation +	Attachment + Proliferation + Differentiation −	Attachment + Proliferation + Differentiation +	Attachment + Proliferation + Differentiation +

+: promoted or accelerated; −: suppressed or delayed

## UV photofunctionalization

### Hydrocarbon pellicle

Physicochemical properties such as superficial chemistry, hydrophilicity/hydrophobicity, and electrostatic charge play crucial roles in determining the biocompatibility and bioactivity of biomaterials [101, 114–119]. However, these properties have often been overlooked or studied limitedly due to their invisible nature, assessment difficulties, and interdependency. For instance, impurities on biomaterials significantly affect surface energy, yet detecting such minor elements or contaminants without advanced devices like X-ray photoelectron spectroscopy (XPS) is challenging [120–122]. Furthermore, contaminants, hydrophilicity, and electrostatic changes can vary independently and dependently [94, 123–129]. UV photofunctionalization aims to optimize these physicochemical properties of titanium [130–148].

Titanium surfaces readily and inevitably adsorb organic molecules from the ambient atmosphere [149, 150], such that ordinary titanium surfaces, regardless of whether they are experimental specimens or commercial implant devices, are covered with hydrocarbon molecules [63, 151–153]. The hydrocarbon layer, or implant pellicle, is significant, as it contaminates bioinert titanium surfaces [123, 154–156]. The pellicle develops over time to increase the atomic percentage of surface carbon (detected by X-ray photoelectron spectroscopy; XPS) by 40–55% and up to over 75% depending on the age of titanium and the surface finish and storage conditions [140, 149, 150, 152, 157]. XPS samples to within 10 nm, within which the layer of passive titanium oxide formed at room temperature measures ∼ 5.5 nm. Therefore, it is reasonable to estimate that the hydrocarbon layer is 4–5 nm thick, which is consistent with the reported atomic percentage of carbon. Thus, the hydrocarbon pellicle is as thick or dense as titanium oxide and should not be ignored, as both osteoblast and bone attachment are reduced on pellicled surfaces. One non-topographical strategy to mitigate the challenges of osteoblasts and the drawback of microrough surfaces could be to “cleanse” the surface and remove hydrocarbon pellicles. UV photofunctionalization has proven to be an effective means to decrease hydrocarbon contamination, making titanium surfaces molecularly clean and genuine by removing the hydrocarbon pellicle [158–160]. Additionally, UV-treated surfaces become biomimetically super-hydrophilic [161–164], thereby promoting cell attachment and proliferation while maintaining differentiation.

### UV-mediated removal of the hydrocarbon pellicle

**Fig. 7:**
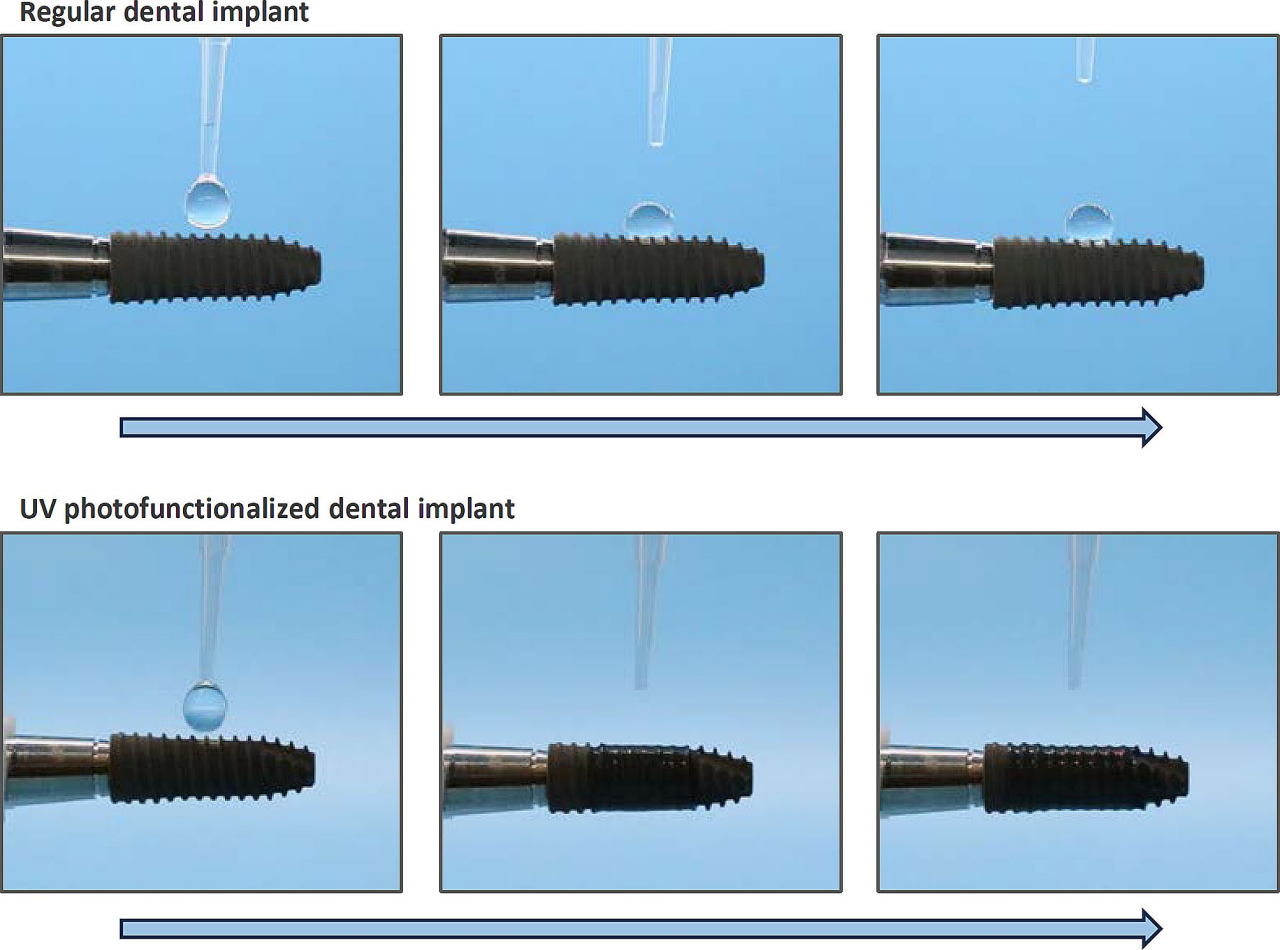
UV photofunctionalization as a physicochemical strategy to overcome the drawback of microrough titanium surfaces. Hydrophobicity/hydrophilicity state of dental implants with or without UV photofunctionalization. Sequential, side-view images depict the behavior of 5 μl ddH_2_O placed on the implants. The implant surfaces are prepared through sandblasting and acid-etching techniques. UV photofunctionalization involves a 1-minute treatment using vacuum UV (VUV) light with a wavelength of 172 nm

UV-mediated removal of the hydrocarbon pellicle, known as UV photofunctionalization, involves treating titanium with UV light immediately prior to use. This method was developed to decompose and eliminate hydrocarbons present on contaminated titanium surfaces, revealing osteoconductive titanium oxide layers [133, 134, 164–175]. Hydrocarbons can be decomposed via three mechanisms: (1) ozone-mediated or non-mediated photochemical decomposition; (2) photophysical decomposition by UV light energy; (3) photocatalytic decomposition induced by titanium dioxide-UV interaction, where 1) and 3) are carried out via the production of reactive oxygen species (ROS) that attack hydrocarbons [155, 176]. As a secondary effect following carbon removal [123], UV photofunctionalized titanium surfaces turn from hydrophobic to superhydrophilic as demonstrated in Fig. 7, fulfilling the two goals of biomimetically hydrophilic and molecularly biocompatible. Initially, UV photofunctionalization required 48-hour exposure of titanium specimens during the early stages of research [135]. However, advancements in technology have reduced this exposure time to 20 min or even 12 min [7, 13, 14, 93, 104, 126, 137, 140, 177–179]. More recently, the next generation of UV photofunctionalization employs high-energy vacuum UV (VUV) light with a wavelength of 172 nm, proving effective with just 1 min of exposure [123, 155, 156, 176, 180] (Fig. 7).

### Biological impact

Numerous in vitro studies have been conducted to assess the biological impact of UV photofunctionalization on osteoblasts, with the hypothesis that it enhances initial interactions between titanium and cells, leading to increased cell attachment and proliferation. Notably, on day 5 of culture, the number of osteoblasts growing on machined titanium surfaces was three times greater than that on acid-etched microrough surfaces [42]. V photofunctionalization augmented the number of osteoblasts on both machined and microrough surfaces, resulting in a cell count on UV-treated microrough surfaces that nearly matched that on untreated machined surfaces [42], effectively offsetting the disadvantage of microrough surfaces. Increased cell proliferation was also confirmed by elevated BrdU incorporation into DNA [42]. Additionally, UV photofunctionalization induced cellular spreading and cytoplasmic projections in vigorously proliferating cells [42, 135]. The rate of differentiation was slightly improved by the increased intercellular interactions due to the accelerated colonization and clustering [42, 135]. Consequently, UV-photofunctionalized titanium achieved robust osseointegration (98.2%) in animal models, nearing complete bone-implant contact [42]. Moreover, mechanically, osseointegration was three times stronger for UV-treated implants at week two, during the early healing period [42].

In a rabbit study, UV photofunctionalization was shown to increase bone-implant contact from 57.8 to 88.4% and double the bone volume [171]. Osteoblast recruitment to UV-treated sites through micropores increased eightfold [40] and it required 42 times the energy to delaminate osteoblasts from the surfaces [21, 41]. Additionally, protein adsorption to titanium increased two to five times on photofunctionalized surfaces Computational fluid dynamics (CFD) studies demonstrated that the amount of blood and proteins carried to microrough implant surfaces is less than half that carried to smooth implant surfaces [181]. However, when the microrough surfaces are hydrophilic, the flow of blood and proteins is doubled, fully offsetting the disadvantage of microrough surfaces These ancillary biological effects collectively contributed to the increased attachment, behavior, and proliferation of osteoblasts, thereby overcoming the osteoblast-rough surface dilemma. The enhanced osteoblastic proliferation and differentiation induced by UV photofunctionalization are supported by clinical outcomes demonstrating reduced healing time [7], accelerated establishment of implant anchorage [182–184], increased success rate [139], and expanded indication of implant therapy [138, 143, 177].

### UV photofunctionalization on different titanium surfaces and devices

UV photofunctionalization has demonstrated effectiveness not only on acid-etched microrough titanium surfaces but also on other titanium surfaces with varying roughness characteristics [104, 130]. For instance, sandblasted surfaces with relatively large-scale roughness compared to acid-etched microrough surfaces have shown increased osteoblast recruitment after UV photofunctionalization [130], with maintained or slightly increased rates of differentiation. Furthermore, UV photofunctionalization has been explored in experimental and clinical applications involving other titanium devices. Titanium mesh plates with microrough surfaces exhibited enhanced osteoblastic attachment following UV treatment without impeding differentiation in vitro, suggesting its potential for guided bone regeneration [34, 185–189]. Similarly, titanium microfibers with microrough textures showed increased osteoblast attraction and attachment upon UV photofunctionalization compared to untreated controls, offering a promising avenue for titanium-driven bone engineering [126, 179, 190].

Moreover, UV photofunctionalization has been found effective on zirconia [134, 156, 172, 175, 191], titanium alloy [140, 164], TiNi [192], and chromium-cobalt alloy [136]. This broad applicability underscores the versatility and potential of UV photofunctionalization as a surface modification technique for enhancing the biocompatibility and bioactivity of various dental and orthopedic implant materials beyond titanium surfaces.

### Utilizing fresh titanium

Freshly surfaced titanium has been demonstrated to exhibit greater bioactivity compared to older titanium surfaces, although the precise mechanisms underlying this phenomenon remain unclear. One hypothesis suggests that newer titanium surfaces, having been exposed to the environment for a shorter duration, accumulate fewer hydrocarbons [142, 151]. In a series of in vitro and in vivo studies, freshly acid-etched titanium disks exhibited minimal carbon deposition; the atomic percentages of carbon were 16% and 62% on new and four-week-old titanium surfaces, respectively [151]. Additionally, freshly prepared microrough titanium surfaces displayed extensive hydrophilic properties [63, 136, 151, 152, 193].

On day 5 of culture, 60% fewer osteoblasts were observed on four-week-old acid-etched microrough titanium surfaces compared to similarly aged machined surfaces. However, 30% more osteoblasts were present on the new acid-etched microrough titanium surfaces than on the four-week-old machined surfaces, indicating that the age of the titanium surface has a greater impact on proliferation than surface roughness [151]. Functional differentiation of osteoblasts, assessed by ALP production and matrix mineralization, was not compromised despite the increased cell proliferation on fresh surfaces. Similar observations were noted for sandblasted, microrough titanium surfaces, and sandblasted CoCr alloy surfaces [136, 151].

Fresh surfacing of titanium surfaces has been shown to increase cell attachment, proliferation, and recruitment and increase adsorption of cell-binding RGD-containing proteins such as fibronectin [152]. These results suggest the new titanium surfaces that are hydrocarbon pellicle-free exhibit a similar effect to UV photofunctionalized titanium surfaces in overcoming the biological dilemmas of osteoblasts and microrough surfaces, thereby re-validating UV photofunctionalization as a novel, effective measure to re-activate old implants, literally transforming old implants to new implants.

Maintaining the freshness of titanium surfaces has been attempted by storing acid-etched titanium specimens in NaCl solution to prevent hydrocarbon pellicle accumulation and preserve surface hydrophilicity [101, 103]. Saline-submerged titanium specimens exhibited surface carbon content of less than 20%; however, the storage duration was not reported [194]. The surface was hydrophilic when examined after drying under nitrogen gas. Nonetheless, the number of osteoblasts attached to NaCl-submerged titanium surfaces was significantly lower than that of control surfaces [54], while the differentiation markers were increased [101, 195], suggesting the effect of surface decarbonization and hydrophilicity can not be generalized.

Thus, strategies to prevent hydrocarbon pellicle formation can be considered anti-aging approaches for titanium implants. Coating freshly acid-etched surfaces with HEPES, a nonvolatile buffer, maintained their superhydrophilicity for at least 3 months and resulted in continuous retention of bioactivity and osteoconductivity comparable to freshly prepared surfaces [196]. This anti-aging coating also served as a drug delivery vehicle; indeed, delivery of an antioxidant amino acid derivative, N-acetyl cysteine (NAC), via HEPES synergistically enhanced the osteoconductivity of the anti-aging titanium surface [196]. Furthermore, aforementioned micro-tempering using nanonodular texturing may exert anti-aging effects on titanium. A study revealed that 7-day-old micro-and-nano hybrid surfaces recruited the same quantity of osteoblasts as freshly prepared surfaces, while 7-day-old microrough surfaces attracted fewer cells compared to their fresh counterparts [94].

### Synergy of UV photofunctionalization and microrough tempering

After demonstrating the individual effects of nano-nodular structuring and UV photofunctionalization in alleviating the biological challenges posed by the inverse osteoblast kinetics on rough surfaces, the hypothesis of combining these two technologies was tested. Osteoblasts were cultured on nano-micro hybrid titanium surfaces with various sizes of nanonodules, with or without UV photofunctionalization [93]. The number of osteoblasts recruited and attached to the hybrid surfaces was significantly greater than on surfaces with micro-pits alone, a phenomenon further amplified by UV photofunctionalization. Hybrid structuring increased recruitment and attachment by 2-4-fold, and UV photofunctionalization enhanced this effect by an additional 2-4-fold, indicating a synergistic interaction [93].

Interestingly, regression analyses revealed a positive correlation between the effect of UV photofunctionalization and the increase in surface area due to nano-texturing, highlighting the beneficial impact of surface tempering on the efficacy of UV photofunctionalization, particularly when coupled with increased surface area [93]. Moreover, the impact of UV photofunctionalization was found to be greater than that of nanonodular texturing [178]. Although nano-structuring was optimized to maximize susceptibility to UV photofunctionalization, the extensive benefits conferred by UV photofunctionalization overshadowed the variable effects of nanotexturing.

## Conclusion

The inverse correlation between osteoblastic proliferation and differentiation on roughened surfaces has been a profound challenge to developing next-generation titanium implant surfaces. Microrough surfaces, the most commonly used surface in dental implants, promote rapid but thin bone formation, whereas machined, smooth surfaces facilitate slower but more voluminous bone formation. In vitro, osteoblast differentiation accelerates but proliferation slows on microrough surfaces, with significantly compromised attachment and initial spreading behavior. This review focused on nano-nodular texturing, meso-structuring, and UV photofunctionalization as prospective solutions to this problem. Nano-nodular texturing of microrough titanium tempers and rounds sharp peaks and edges while preserving the primary micro-pit configuration. Despite an increase in surface roughness, osteoblast attachment and proliferation are significantly amplified on nanotextured, micro-tempered surfaces, with osteoblasts demonstrating enhanced differentiation. Meso-texturing involves creating meso-scale (> 10 μm) spikes with typical micro-pits that effectively increase implant surface area, allowing more osteoblasts to attach and proliferate on the meso-and-micro hybrid surfaces compared with microrough surfaces, counteracting the drawbacks of rougher surfaces. UV photofunctionalization purifies surfaces by eliminating the hydrocarbon pellicle that inevitably accumulates on titanium surfaces over time, transforming surfaces into biomimetically hydrophilic entities. Photofunctionalized microrough surfaces increase cell attachment and proliferation while maintaining accelerated differentiation. These topographical and physicochemical strategies effectively alleviate and even resolve the biological challenges of osteoblast kinetics and rough surfaces and now need full exploitation and implementation for the development of next-generation implants.

## Data Availability

No datasets were generated or analysed during the current study.

## Abbreviations

BIC: Bone-implant contact
CFD: Computational fluid dynamic
HA: Hydroxyapatite
NAC: N-acetyl cysteine
PCNA: Proliferating cell nuclear antigen
PLA: Poly-lactic acid
SEM: Scanning electron microscopy
TPS: Titanium plasma sprayed
VUV: Vacuum UV
XPS: X-ray photoelectron spectroscopy
Y-TZP: Yttria-stabilized tetragonal zirconia polycrystal

## References

[1] Cooper LF (1998). Biologic determinants of bone formation for osseointegration: clues for future clinical improvements. J Prosthet Dent.

[2] Masuda T, Yliheikkila PK, Felton DA, Cooper LF (1998). Generalizations regarding the process and phenomenon of osseointegration. Part I. In vivo studies. Int J Oral Maxillofac Implants.

[3] Stanford CM (1999). Biomechanical and functional behavior of implants. Adv Dent Res.

[4] Ogawa T, Nishimura I (2003). Different bone integration profiles of turned and acid-etched implants associated with modulated expression of extracellular matrix genes. Int J Oral Maxillofac Implants.

[5] Albrektsson T, Wennerberg A (2019). On osseointegration in relation to implant surfaces. Clin Implant Dent Relat Res.

[6] Wennerberg A, Albrektsson T (2009). Effects of titanium surface topography on bone integration: a systematic review. Clin Oral Implants Res.

[7] Funato A, Yamada M, Ogawa T (2013). Success rate, healing time, and implant stability of photofunctionalized dental implants. Int J Oral Maxillofac Implants.

[8] Almassri HNS, Ma Y, Dan Z, Ting Z, Cheng Y, Wu X (2020). Implant stability and survival rates of a hydrophilic versus a conventional sandblasted, acid-etched implant surface: systematic review and meta-analysis. J Am Dent Assoc.

[9] Chambrone L, Rincon-Castro MV, Poveda-Marin AE, Diazgranados-Lozano MP, Fajardo-Escolar CE, Bocanegra-Puerta MC (2020). Histological healing outcomes at the bone-titanium interface of loaded and unloaded dental implants placed in humans: a systematic review of controlled clinical trials. Int J Oral Implantol (Berl).

[10] Giro G, Chambrone L, Goldstein A, Rodrigues JA, Zenobio E, Feres M (2015). Impact of osteoporosis in dental implants: a systematic review. World J Orthop.

[11] Chrcanovic BR, Albrektsson T, Wennerberg A (2015). Smoking and dental implants: a systematic review and meta-analysis. J Dent.

[12] Ishijima M, Ghassemi A, Soltanzadeh P, Tanaka M, Nakhaei K, Park W (2016). Effect of UV photofunctionalization on osseointegration in aged rats. Implant Dent.

[13] Taniyama T, Saruta J, Mohammadzadeh Rezaei N, Nakhaei K, Ghassemi A, Hirota M et al. UV-photofunctionalization of titanium promotes mechanical anchorage in a rat osteoporosis model. Int J Mol Sci. 2020;21(4). 10.3390/ijms21041235 PMC7072956 32059603

[14] Sugita Y, Honda Y, Kato I, Kubo K, Maeda H, Ogawa T (2014). Role of photofunctionalization in mitigating impaired osseointegration associated with type 2 diabetes in rats. Int J Oral Maxillofac Implants.

[15] Hasegawa H, Ozawa S, Hashimoto K, Takeichi T, Ogawa T (2008). Type 2 diabetes impairs implant osseointegration capacity in rats. Int J Oral Maxillofac Implants.

[16] Ting M, Huynh BH, Woldu HG, Gamal I, Suzuki JB. Clinical impact on dental implant survival in patients taking antiresorptive medications: a systematic review and meta-analysis. J Oral Implantol. 2023. 10.1563/aaid-joi-D-21-00160 37905745

[17] Oh SL, Shiau HJ, Reynolds MA (2020). Survival of dental implants at sites after implant failure: a systematic review. J Prosthet Dent.

[18] Howe MS, Keys W, Richards D (2019). Long-term (10-year) dental implant survival: a systematic review and sensitivity meta-analysis. J Dent.

[19] Goyal S, Masood M, Le C, Rajendran Y, Nanjapa S, Vaderhobli R (2021). Comparative bone graft evaluation for Dental Implant Success: an evidence-based review. J Long Term Eff Med Implants.

[20] Weinlaender M, Kenney EB, Lekovic V, Beumer J, Moy PK, Lewis S (1992). Histomorphometry of bone apposition around three types of endosseous dental implants. Int J Oral Maxillofac Implants.

[21] Berglundh T, Abrahamsson I, Albouy JP, Lindhe J (2007). Bone healing at implants with a fluoride-modified surface: an experimental study in dogs. Clin Oral Implants Res.

[22] De Maeztu MA, Braceras I, Alava JI, Gay-Escoda C (2008). Improvement of osseointegration of titanium dental implant surfaces modified with CO ions: a comparative histomorphometric study in beagle dogs. Int J Oral Maxillofac Surg.

[23] Albrektsson T, Wennerberg A (2004). Oral implant surfaces: part 2–review focusing on clinical knowledge of different surfaces. Int J Prosthodont.

[24] Albrektsson T, Wennerberg A (2004). Oral implant surfaces: part 1–review focusing on topographic and chemical properties of different surfaces and in vivo responses to them. Int J Prosthodont.

[25] Jokstad A, Braegger U, Brunski JB, Carr AB, Naert I, Wennerberg A (2003). Quality of dental implants. Int Dent J.

[26] Lopez-Valverde N, Flores-Fraile J, Ramirez JM, Sousa BM, Herrero-Hernandez S, Lopez-Valverde A. Bioactive surfaces vs. conventional surfaces in titanium dental implants: a comparative systematic review. J Clin Med. 2020;9(7). 10.3390/jcm9072047 PMC7408888 32610687

[27] Chambrone L, Shibli JA, Mercurio CE, Cardoso B, Preshaw PM (2015). Efficacy of standard (SLA) and modified sandblasted and acid-etched (SLActive) dental implants in promoting immediate and/or early occlusal loading protocols: a systematic review of prospective studies. Clin Oral Implants Res.

[28] Yeo IS (2014). Reality of dental implant surface modification: a short literature review. Open Biomed Eng J.

[29] Stadlinger B, Pourmand P, Locher MC, Schulz MC (2012). Systematic review of animal models for the study of implant integration, assessing the influence of material, surface and design. J Clin Periodontol.

[30] Matos GRM (2021). Surface roughness of Dental Implant and Osseointegration. J Maxillofac Oral Surg.

[31] Boyan BD, Bonewald LF, Paschalis EP, Lohmann CH, Rosser J, Cochran DL (2002). Osteoblast-mediated mineral deposition in culture is dependent on surface microtopography. Calcif Tissue Int.

[32] Buser D, Nydegger T, Oxland T, Cochran DL, Schenk RK, Hirt HP (1999). Interface shear strength of titanium implants with a sandblasted and acid-etched surface: a biomechanical study in the maxilla of miniature pigs. J Biomed Mater Res.

[33] Stafford GL (2014). Review found little difference between sandblasted and acid-etched (SLA) dental implants and modified surface (SLActive) implants. Evid Based Dent.

[34] Saruta J, Sato N, Ishijima M, Okubo T, Hirota M, Ogawa T. Disproportionate effect of sub-micron topography on osteoconductive capability of titanium. Int J Mol Sci. 2019;20(16). 10.3390/ijms20164027 PMC6720784 31426563

[35] Att W, Tsukimura N, Suzuki T, Ogawa T (2007). Effect of supramicron roughness characteristics produced by 1- and 2-step acid etching on the osseointegration capability of titanium. Int J Oral Maxillofac Implants.

[36] Takeuchi K, Saruwatari L, Nakamura HK, Yang JM, Ogawa T (2005). Enhanced intrinsic biomechanical properties of osteoblastic mineralized tissue on roughened titanium surface. J Biomed Mater Res A.

[37] Tsukimura N, Kojima N, Kubo K, Att W, Takeuchi K, Kameyama Y (2008). The effect of superficial chemistry of titanium on osteoblastic function. J Biomed Mater Res A.

[38] Ozawa S, Ogawa T, Iida K, Sukotjo C, Hasegawa H, Nishimura RD (2002). Ovariectomy hinders the early stage of bone-implant integration: histomorphometric, biomechanical, and molecular analyses. Bone.

[39] Butz F, Aita H, Wang CJ, Ogawa T (2006). Harder and stiffer bone osseointegrated to roughened titanium. J Dent Res.

[40] Hasegawa M, Saruta J, Hirota M, Taniyama T, Sugita Y, Kubo K et al. A newly created meso-, micro-, and nano-scale rough titanium surface promotes bone-implant integration. Int J Mol Sci. 2020;21(3). 10.3390/ijms21030783 PMC7036846 31991761

[41] Saruwatari L, Aita H, Butz F, Nakamura HK, Ouyang J, Yang Y (2005). Osteoblasts generate harder, stiffer, and more delamination-resistant mineralized tissue on titanium than on polystyrene, associated with distinct tissue micro- and ultrastructure. J Bone Min Res.

[42] Aita H, Hori N, Takeuchi M, Suzuki T, Yamada M, Anpo M (2009). The effect of ultraviolet functionalization of titanium on integration with bone. Biomaterials.

[43] Park G, Matsuura T, Komatsu K, Ogawa T. Optimizing implant osseointegration, soft tissue responses, and bacterial inhibition: a comprehensive narrative review on the multifaceted approach of the UV photofunctionalization of titanium. J Prosthodont Res. in press. 10.2186/jpr.JPR_D_24_00086 38853001

[44] Komatsu K, Matsuura T, Suzumura T, Ogawa T (2023). Genome-wide transcriptional responses of osteoblasts to different titanium surface topographies. Mater Today Bio.

[45] Billiard J, Moran RA, Whitley MZ, Chatterjee-Kishore M, Gillis K, Brown EL (2003). Transcriptional profiling of human osteoblast differentiation. J Cell Biochem.

[46] Stein GS, Lian JB (1993). Molecular mechanisms mediating proliferation/differentiation interrelationships during progressive development of the osteoblast phenotype. Endocr Rev.

[47] Owen TA, Aronow M, Shalhoub V, Barone LM, Wilming L, Tassinari MS (1990). Progressive development of the rat osteoblast phenotype in vitro: reciprocal relationships in expression of genes associated with osteoblast proliferation and differentiation during formation of the bone extracellular matrix. J Cell Physiol.

[48] Siddhanti SR, Quarles LD (1994). Molecular to pharmacologic control of osteoblast proliferation and differentiation. J Cell Biochem.

[49] Alborzi A, Mac K, Glackin CA, Murray SS, Zernik JH (1996). Endochondral and intramembranous fetal bone development: osteoblastic cell proliferation, and expression of alkaline phosphatase, m-twist, and histone H4. J Craniofac Genet Dev Biol.

[50] Hori N, Iwasa F, Ueno T, Takeuchi K, Tsukimura N, Yamada M (2010). Selective cell affinity of biomimetic micro-nano-hybrid structured TiO2 overcomes the biological dilemma of osteoblasts. Dent Materials: Official Publication Acad Dent Mater.

[51] Alliston T, Choy L, Ducy P, Karsenty G, Derynck R (2001). TGF-beta-induced repression of CBFA1 by Smad3 decreases cbfa1 and osteocalcin expression and inhibits osteoblast differentiation. Embo J.

[52] Spinella-Jaegle S, Roman-Roman S, Faucheu C, Dunn FW, Kawai S, Gallea S (2001). Opposite effects of bone morphogenetic protein-2 and transforming growth factor-beta1 on osteoblast differentiation. Bone.

[53] Bachle M, Kohal RJ (2004). A systematic review of the influence of different titanium surfaces on proliferation, differentiation and protein synthesis of osteoblast-like MG63 cells. Clin Oral Implants Res.

[54] Zhao G, Schwartz Z, Wieland M, Rupp F, Geis-Gerstorfer J, Cochran DL (2005). High surface energy enhances cell response to titanium substrate microstructure. J Biomed Mater Res A.

[55] Kitajima H, Komatsu K, Matsuura T, Ozawa R, Saruta J, Taleghani SR (2023). Impact of nano-scale trabecula size on osteoblastic behavior and function in a meso-nano hybrid rough biomimetic zirconia model. J Prosthodont Res.

[56] Rezaei NM, Hasegawa M, Ishijima M, Nakhaei K, Okubo T, Taniyama T (2018). Biological and osseointegration capabilities of hierarchically (meso-/micro-/nano-scale) roughened zirconia. Int J Nanomed.

[57] Saruta J, Ozawa R, Okubo T, Taleghani SR, Ishijima M, Kitajima H et al. Biomimetic zirconia with cactus-inspired meso-scale spikes and nano-trabeculae for enhanced bone integration. Int J Mol Sci. 2021;22(15). 10.3390/ijms22157969 PMC8347469 34360734

[58] Ogawa T, Sukotjo C, Nishimura I (2002). Modulated bone matrix-related gene expression is associated with differences in interfacial strength of different implant surface roughness. J Prosthodont.

[59] Lian JB, Javed A, Zaidi SK, Lengner C, Montecino M, van Wijnen AJ (2004). Regulatory controls for osteoblast growth and differentiation: role of Runx/Cbfa/AML factors. Crit Rev Eukaryot Gene Expr.

[60] Thomas DM, Johnson SA, Sims NA, Trivett MK, Slavin JL, Rubin BP (2004). Terminal osteoblast differentiation, mediated by runx2 and p27KIP1, is disrupted in osteosarcoma. J Cell Biol.

[61] Yang K, Hitomi M, Stacey DW (2006). Variations in cyclin D1 levels through the cell cycle determine the proliferative fate of a cell. Cell Div.

[62] Xiong Y, Zhang H, Beach D (1992). D type cyclins associate with multiple protein kinases and the DNA replication and repair factor PCNA. Cell.

[63] Att W, Ogawa T (2012). Biological aging of implant surfaces and their restoration with ultraviolet light treatment: a novel understanding of osseointegration. Int J Oral Maxillofac Implants.

[64] Davies JE (1996). In vitro modeling of the bone/implant interface. Anat Rec.

[65] Davies JE (2003). Understanding peri-implant endosseous healing. J Dent Educ.

[66] Kikuchi L, Park JY, Victor C, Davies JE (2005). Platelet interactions with calcium-phosphate-coated surfaces. Biomaterials.

[67] Donos N, Retzepi M, Wall I, Hamlet S, Ivanovski S (2011). In vivo gene expression profile of guided bone regeneration associated with a microrough titanium surface. Clin Oral Implants Res.

[68] Butz F, Aita H, Takeuchi K, Ogawa T (2005). Enhanced mineralized tissue adhesion to titanium over polystyrene assessed by the nano-scratch test. J Biomed Mater Res A.

[69] Nakamura H, Shim J, Butz F, Aita H, Gupta V, Ogawa T (2006). Glycosaminoglycan degradation reduces mineralized tissue-titanium interfacial strength. J Biomed Mater Res A.

[70] Nakamura HK, Butz F, Saruwatari L, Ogawa T (2007). A role for proteoglycans in mineralized tissue-titanium adhesion. J Dent Res.

[71] Butz F, Ogawa T, Chang TL, Nishimura I (2006). Three-dimensional bone-implant integration profiling using micro-computed tomography. Int J Oral Maxillofac Implants.

[72] Butz F, Ogawa T, Nishimura I (2011). Interfacial shear strength of endosseous implants. Int J Oral Maxillofac Implants.

[73] Shim J, Nakamura H, Ogawa T, Gupta V (2009). An understanding of the mechanism that promotes adhesion between roughened titanium implants and mineralized tissue. J Biomech Eng.

[74] Ogawa T, Ozawa S, Shih JH, Ryu KH, Sukotjo C, Yang JM (2000). Biomechanical evaluation of osseous implants having different surface topographies in rats. J Dent Res.

[75] Uno M, Hayashi M, Ozawa R, Saruta J, Ishigami H, Ogawa T (2020). Mechanical interlocking capacity of Titanium with respect to Surface morphology and topographical parameters. J Dentistry Oral Biology.

[76] Uno M, Ozawa R, Hamajima K, Saruta J, Ishigami H, Ogawa T (2020). Variation in osteoblast Retention ability of Titanium surfaces with different topographies. J Dentistry Oral Biology.

[77] Sato N, Kubo K, Yamada M, Hori N, Suzuki T, Maeda H (2009). Osteoblast mechanoresponses on Ti with different surface topographies. J Dent Res.

[78] Ogawa T, Nishimura I (2006). Genes differentially expressed in titanium implant healing. J Dent Res.

[79] Kojima N, Ozawa S, Miyata Y, Hasegawa H, Tanaka Y, Ogawa T (2008). High-throughput gene expression analysis in bone healing around titanium implants by DNA microarray. Clin Oral Implants Res.

[80] Tsukimura N, Ueno T, Iwasa F, Minamikawa H, Sugita Y, Ishizaki K (2011). Bone integration capability of alkali- and heat-treated nanobimorphic Ti-15Mo-5Zr-3Al. Acta Biomater.

[81] Ueno T, Tsukimura N, Yamada M, Ogawa T (2011). Enhanced bone-integration capability of alkali- and heat-treated nanopolymorphic titanium in micro-to-nanoscale hierarchy. Biomaterials.

[82] Yamada M, Ueno T, Tsukimura N, Ikeda T, Nakagawa K, Hori N (2012). Bone integration capability of nanopolymorphic crystalline hydroxyapatite coated on titanium implants. Int J Nanomed.

[83] Cooper LF (2000). A role for surface topography in creating and maintaining bone at titanium endosseous implants. J Prosthet Dent.

[84] Cooper LF, Masuda T, Yliheikkila PK, Felton DA (1998). Generalizations regarding the process and phenomenon of osseointegration. Part II. In vitro studies. Int J Oral Maxillofac Implants.

[85] Cooper LF, Zhou Y, Takebe J, Guo J, Abron A, Holmen A (2006). Fluoride modification effects on osteoblast behavior and bone formation at TiO2 grit-blasted c.p. titanium endosseous implants. Biomaterials.

[86] Guo J, Padilla RJ, Ambrose W, De Kok IJ, Cooper LF (2007). The effect of hydrofluoric acid treatment of TiO2 grit blasted titanium implants on adherent osteoblast gene expression in vitro and in vivo. Biomaterials.

[87] Kubo K, Att W, Yamada M, Ohmi K, Tsukimura N, Suzuki T (2008). Microtopography of titanium suppresses osteoblastic differentiation but enhances chondroblastic differentiation of rat femoral periosteum-derived cells. J Biomedical Mater Res Part A.

[88] Annunziata M, Oliva A, Basile MA, Giordano M, Mazzola N, Rizzo A (2011). The effects of titanium nitride-coating on the topographic and biological features of TPS implant surfaces. J Dent.

[89] Gottlander M, Albrektsson T (1992). Histomorphometric analyses of hydroxyapatite-coated and uncoated titanium implants. The importance of the implant design. Clin Oral Implants Res.

[90] Jokstad A, Sanz M, Ogawa T, Bassi F, Levin L, Wennerberg A (2016). A systematic review of the role of Implant Design in the Rehabilitation of the Edentulous Maxilla. Int J Oral Maxillofac Implants.

[91] Kubo K, Tsukimura N, Iwasa F, Ueno T, Saruwatari L, Aita H (2009). Cellular behavior on TiO2 nanonodular structures in a micro-to-nanoscale hierarchy model. Biomaterials.

[92] Ogawa T, Saruwatari L, Takeuchi K, Aita H, Ohno N (2008). Ti Nano-nodular structuring for bone integration and regeneration. J Dent Res.

[93] Tsukimura N, Yamada M, Iwasa F, Minamikawa H, Att W, Ueno T (2011). Synergistic effects of UV photofunctionalization and micro-nano hybrid topography on the biological properties of titanium. Biomaterials.

[94] Iwasa F, Tsukimura N, Sugita Y, Kanuru RK, Kubo K, Hasnain H (2011). TiO2 micro-nano-hybrid surface to alleviate biological aging of UV-photofunctionalized titanium. Int J Nanomed.

[95] Bays JL, DeMali KA (2017). Vinculin in cell-cell and cell-matrix adhesions. Cell Mol Life Sci.

[96] Atherton P, Stutchbury B, Jethwa D, Ballestrem C (2016). Mechanosensitive components of integrin adhesions: role of vinculin. Exp Cell Res.

[97] Rothenberg KE, Scott DW, Christoforou N, Hoffman BD (2018). Vinculin Force-Sensitive Dynamics at Focal adhesions Enable Effective Directed Cell Migration. Biophys J.

[98] Case LB, Baird MA, Shtengel G, Campbell SL, Hess HF, Davidson MW (2015). Molecular mechanism of vinculin activation and nanoscale spatial organization in focal adhesions. Nat Cell Biol.

[99] Wennerberg A, Galli S, Albrektsson T (2011). Current knowledge about the hydrophilic and nanostructured SLActive surface. Clin Cosmet Invest Dentistry.

[100] Dohan Ehrenfest DM, Vazquez L, Park YJ, Sammartino G, Bernard JP (2011). Identification card and codification of the chemical and morphological characteristics of 14 dental implant surfaces. J Oral Implantol.

[101] Zhao G, Raines AL, Wieland M, Schwartz Z, Boyan BD (2007). Requirement for both micron- and submicron scale structure for synergistic responses of osteoblasts to substrate surface energy and topography. Biomaterials.

[102] Olivares-Navarrete R, Raz P, Zhao G, Chen J, Wieland M, Cochran DL (2008). Integrin alpha2beta1 plays a critical role in osteoblast response to micron-scale surface structure and surface energy of titanium substrates. Proc Natl Acad Sci U S A.

[103] Gittens RA, Scheideler L, Rupp F, Hyzy SL, Geis-Gerstorfer J, Schwartz Z (2014). A review on the wettability of dental implant surfaces II: Biological and clinical aspects. Acta Biomater.

[104] Ikeda T, Hagiwara Y, Hirota M, Tabuchi M, Yamada M, Sugita Y (2014). Effect of photofunctionalization on fluoride-treated nanofeatured titanium. J Biomater Appl.

[105] Sul YT, Johansson CB, Jeong Y, Roser K, Wennerberg A, Albrektsson T (2001). Oxidized implants and their influence on the bone response. J Mater Sci Mater Med.

[106] Wurihan, Yamada A, Suzuki D, Shibata Y, Kamijo R, Miyazaki T (2015). Enhanced in vitro biological activity generated by surface characteristics of anodically oxidized titanium–the contribution of the oxidation effect. Eur Cell Mater.

[107] Echeverry-Rendon M, Galvis O, Quintero Giraldo D, Pavon J, Lopez-Lacomba JL, Jimenez-Pique E (2015). Osseointegration improvement by plasma electrolytic oxidation of modified titanium alloys surfaces. J Mater Sci Mater Med.

[108] Sugita Y, Ishizaki K, Iwasa F, Ueno T, Minamikawa H, Yamada M (2011). Effects of pico-to-nanometer-thin TiO2 coating on the biological properties of microroughened titanium. Biomaterials.

[109] Wang Q, Zhou P, Liu S, Attarilar S, Ma RL, Zhong Y et al. Multi-scale surface treatments of titanium implants for rapid osseointegration: a review. Nanomaterials (Basel). 2020;10(6). 10.3390/nano10061244 PMC7353126 32604854

[110] Borges H, Correia ARM, Castilho RM, de Oliveira Fernandes GV (2020). Zirconia implants and marginal bone loss: a systematic review and Meta-analysis of Clinical studies. Int J Oral Maxillofac Implants.

[111] Depprich R, Zipprich H, Ommerborn M, Naujoks C, Wiesmann HP, Kiattavorncharoen S (2008). Osseointegration of zirconia implants compared with titanium: an in vivo study. Head Face Med.

[112] Elnayef B, Lazaro A, Suarez-Lopez Del Amo F, Galindo-Moreno P, Wang HL, Gargallo-Albiol J et al. Zirconia implants as an alternative to titanium: a systematic review and meta-analysis. Int J Oral Maxillofac Implants. 2017. 10.11607/jomi.5223 28170450

[113] Hashim D, Cionca N, Courvoisier DS, Mombelli A (2016). A systematic review of the clinical survival of zirconia implants. Clin Oral Investig.

[114] Dorfman JD (1986). Surface energy effects of implant biomaterials on the implant-tissue interface: implications for the rate, character and quality of post-surgical healing. J Oral Implantol.

[115] dos Santos EA, Farina M, Soares GA, Anselme K (2008). Surface energy of hydroxyapatite and beta-tricalcium phosphate ceramics driving serum protein adsorption and osteoblast adhesion. J Mater Sci Mater Med.

[116] Hempel U, Hefti T, Dieter P, Schlottig F (2013). Response of human bone marrow stromal cells, MG-63, and SaOS-2 to titanium-based dental implant surfaces with different topography and surface energy. Clin Oral Implants Res.

[117] Lai HC, Zhuang LF, Liu X, Wieland M, Zhang ZY, Zhang ZY (2010). The influence of surface energy on early adherent events of osteoblast on titanium substrates. J Biomed Mater Res A.

[118] Sajjady SA, Lotfi M, Amini S, Toutounchi H, Bami AB (2019). Improving the surface energy of titanium implants by the creation of hierarchical textures on the surface via three-dimensional elliptical vibration turning for enhanced osseointegration. Proc Inst Mech Eng H.

[119] Uchiyama H, Yamada M, Ishizaki K, Sakurai K (2014). Specific ultraviolet-C irradiation energy for functionalization of titanium surface to increase osteoblastic cellular attachment. J Biomater Appl.

[120] Hao S, Wang M, Yin Z, Jing Y, Bai L, Su J (2023). Microenvironment-targeted strategy steers advanced bone regeneration. Mater Today Bio.

[121] Duddeck DU, Albrektsson T, Wennerberg A, Larsson C, Beuer F. On the cleanliness of different oral implant systems: a pilot study. J Clin Med. 2019;8(9). 10.3390/jcm8091280 PMC6780125 31443535

[122] Mayer BK, Johnson C, Yang Y, Wellenstein N, Maher E, McNamara PJ (2019). From micro to macro-contaminants: the impact of low-energy titanium dioxide photocatalysis followed by filtration on the mitigation of drinking water organics. Chemosphere.

[123] Kido D, Komatsu K, Suzumura T, Matsuura T, Cheng J, Kim J (2023). Influence of Surface contaminants and Hydrocarbon Pellicle on the results of wettability measurements of Titanium. Int J Mol Sci.

[124] Iwasa F, Baba K, Ogawa T (2016). Enhanced intracellular signaling pathway in osteoblasts on ultraviolet lighttreated hydrophilic titanium. Biomed Res.

[125] Iwasa F, Hori N, Ueno T, Minamikawa H, Yamada M, Ogawa T (2010). Enhancement of osteoblast adhesion to UV-photofunctionalized titanium via an electrostatic mechanism. Biomaterials.

[126] Iwasaki C, Hirota M, Tanaka M, Kitajima H, Tabuchi M, Ishijima M et al. Tuning of titanium microfiber scaffold with UV-photofunctionalization for enhanced osteoblast affinity and function. Int J Mol Sci. 2020;21(3). 10.3390/ijms21030738 PMC7036837 31979313

[127] Miyauchi T, Yamada M, Yamamoto A, Iwasa F, Suzawa T, Kamijo R (2010). The enhanced characteristics of osteoblast adhesion to photofunctionalized nanoscale TiO2 layers on biomaterials surfaces. Biomaterials.

[128] Ueno T, Takeuchi M, Hori N, Iwasa F, Minamikawa H, Igarashi Y (2012). Gamma ray treatment enhances bioactivity and osseointegration capability of titanium. J Biomedical Mater Res Part B Appl Biomaterials.

[129] Yamada M, Miyauchi T, Yamamoto A, Iwasa F, Takeuchi M, Anpo M (2010). Enhancement of adhesion strength and cellular stiffness of osteoblasts on mirror-polished titanium surface by UV-photofunctionalization. Acta Biomater.

[130] Suzuki T, Hori N, Att W, Kubo K, Iwasa F, Ueno T (2009). Ultraviolet treatment overcomes time-related degrading bioactivity of titanium. Tissue Eng Part A.

[131] Tabuchi M, Hamajima K, Tanaka M, Sekiya T, Hirota M, Ogawa T. UV light-generated superhydrophilicity of a titanium surface enhances the transfer, diffusion and adsorption of osteogenic factors from a collagen sponge. Int J Mol Sci. 2021;22(13). 10.3390/ijms22136811 PMC8268603 34202795

[132] Sugita Y, Saruta J, Taniyama T, Kitajima H, Hirota M, Ikeda T et al. UV-pre-treated and protein-adsorbed titanium implants exhibit enhanced osteoconductivity. Int J Mol Sci. 2020;21(12). 10.3390/ijms21124194 PMC7349557 32545509

[133] Chang LC. Clinical applications of photofunctionalization on dental implant surfaces: a narrative review. J Clin Med. 2022;11(19). 10.3390/jcm11195823 PMC9571244 36233693

[134] Flanagan D (2016). Photofunctionalization of Dental implants. J Oral Implantol.

[135] Aita H, Att W, Ueno T, Yamada M, Hori N, Iwasa F (2009). Ultraviolet light-mediated photofunctionalization of titanium to promote human mesenchymal stem cell migration, attachment, proliferation and differentiation. Acta Biomater.

[136] Att W, Hori N, Iwasa F, Yamada M, Ueno T, Ogawa T (2009). The effect of UV-photofunctionalization on the time-related bioactivity of titanium and chromium-cobalt alloys. Biomaterials.

[137] de Avila ED, Lima BP, Sekiya T, Torii Y, Ogawa T, Shi W (2015). Effect of UV-photofunctionalization on oral bacterial attachment and biofilm formation to titanium implant material. Biomaterials.

[138] Hirota M, Ozawa T, Iwai T, Mitsudo K, Ogawa T. UV-mediated photofunctionalization of dental implant: a seven-year results of a prospective study. J Clin Med. 2020;9(9). 10.3390/jcm9092733 PMC7565265 32847061

[139] Hirota M, Ozawa T, Iwai T, Ogawa T, Tohnai I (2018). Effect of photofunctionalization on early Implant failure. Int J Oral Maxillofac Implants.

[140] Minamikawa H, Ikeda T, Att W, Hagiwara Y, Hirota M, Tabuchi M (2014). Photofunctionalization increases the bioactivity and osteoconductivity of the titanium alloy Ti6Al4V. J Biomed Mater Res A.

[141] Ogawa T (2012). UV-photofunctionalization of titanium implants. Oral Craniofac Tissue Eng.

[142] Pyo SW, Park YB, Moon HS, Lee JH, Ogawa T (2013). Photofunctionalization enhances bone-implant contact, dynamics of interfacial osteogenesis, marginal bone seal, and removal torque value of implants: a dog jawbone study. Implant Dent.

[143] Hirota M, Ozawa T, Iwai T, Ogawa T, Tohnai I (2016). Implant Stability Development of Photofunctionalized Implants Placed in regular and complex cases: a case-control study. Int J Oral Maxillofac Implants.

[144] Att W, Takeuchi M, Suzuki T, Kubo K, Anpo M, Ogawa T (2009). Enhanced osteoblast function on ultraviolet light-treated zirconia. Biomaterials.

[145] Okubo T, Ikeda T, Saruta J, Tsukimura N, Hirota M, Ogawa T (2020). Compromised epithelial cell attachment after polishing Titanium Surface and its restoration by UV treatment. Mater (Basel).

[146] Ueno T, Ikeda T, Tsukimura N, Ishijima M, Minamikawa H, Sugita Y (2016). Novel antioxidant capability of titanium induced by UV light treatment. Biomaterials.

[147] Ueno T, Yamada M, Hori N, Suzuki T, Ogawa T (2010). Effect of ultraviolet photoactivation of titanium on osseointegration in a rat model. Int J Oral Maxillofac Implants.

[148] Ueno T, Yamada M, Suzuki T, Minamikawa H, Sato N, Hori N (2010). Enhancement of bone-titanium integration profile with UV-photofunctionalized titanium in a gap healing model. Biomaterials.

[149] Morra M, Cassinelli C, Bruzzone G, Carpi A, Di Santi G, Giardino R (2003). Surface chemistry effects of topographic modification of titanium dental implant surfaces: 1. Surface analysis. Int J Oral Maxillofac Implants.

[150] Massaro C, Rotolo P, De Riccardis F, Milella E, Napoli A, Wieland M (2002). Comparative investigation of the surface properties of commercial titanium dental implants. Part I: chemical composition. J Mater Sci - Mater Med.

[151] Att W, Hori N, Takeuchi M, Ouyang J, Yang Y, Anpo M (2009). Time-dependent degradation of titanium osteoconductivity: an implication of biological aging of implant materials. Biomaterials.

[152] Hori N, Att W, Ueno T, Sato N, Yamada M, Saruwatari L (2009). Age-dependent degradation of the protein adsorption capacity of titanium. J Dent Res.

[153] Lee JH, Ogawa T (2012). The biological aging of titanium implants. Implant Dent.

[154] Ogawa T. Photofunctionalization of TiO2 for optimal integration of titanium with bone. In Benign Photocatalysts Applications of titanium oxide-based materials Eds Prashant Kamat and Masakazu Anpo Springer US. 2010:699–713.

[155] Suzumura T, Matsuura T, Komatsu K, Ogawa T. Decomposing organic molecules on titanium with vacuum ultraviolet light for effective and rapid photofunctionalization. J Funct Biomater. 2022;14(1). 10.3390/jfb14010011 PMC9861116 36662058

[156] Suzumura T, Matusura T, Komatsu K, Sugita Y, Maeda H, Ogawa T (2023). Vacuum Ultraviolet (VUV) light photofunctionalization to induce human oral fibroblast transmigration on Zirconia. Cells.

[157] Minamikawa H, Att W, Ikeda T, Hirota M, Ogawa T. Long-term progressive degradation of the biological capability of titanium. Materials. 2016;9(102). 10.3390/ma9020102) 10.3390/ma9020102 PMC5456510 28787899

[158] Uosaki K, Quayum ME, Nihonyanagi S, Kondo T (2004). Decomposition processes of an organic monolayer formed on Si(111) via a silicon-carbon bond induced by exposure to UV irradiation or ozone. Langmuir.

[159] Zhang L, Li P, Gong Z, Li X (2008). Photocatalytic degradation of polycyclic aromatic hydrocarbons on soil surfaces using TiO(2) under UV light. J Hazard Mater.

[160] Zubkov T, Stahl D, Thompson TL, Panayotov D, Diwald O, Yates JT (2005). Ultraviolet light-induced hydrophilicity effect on TiO2(110)(1 × 1). Dominant role of the photooxidation of adsorbed hydrocarbons causing wetting by water droplets. J Phys Chem B.

[161] Boyan BD, Lotz EM, Schwartz Z. (*) Roughness and hydrophilicity as osteogenic biomimetic surface properties. Tissue Eng Part A. 2017;23(23–24):1479–89. 10.1089/ten.tea.2017.0048 PMC5729880 28793839

[162] Wang R, Hashimoto K, Fujishima A (1997). Light-induced amphiphilic surfaces. Nature.

[163] Dohshi S, Takeuchi M, Anpo M (2001). Photoinduced superhydrophilic properties of Ti-B binary oxide thin films and their photocatalytic reactivity for the decomposition of NO. J Nanosci Nanotechnol.

[164] Yin C, Zhang T, Wei Q, Cai H, Cheng Y, Tian Y (2022). Surface treatment of 3D printed porous Ti6Al4V implants by ultraviolet photofunctionalization for improved osseointegration. Bioact Mater.

[165] Dini C, Nagay BE, Magno MB, Maia LC, Barao VAR (2020). Photofunctionalization as a suitable approach to improve the osseointegration of implants in animal models-A systematic review and meta-analysis. Clin Oral Implants Res.

[166] Jaikumar RA, Karthigeyan S, Bhat R, Naidu M, Natarajan S, Angamuthu V (2021). Analysis of Surface morphology and elemental composition on Zirconia implants before and after photofunctionalization by Scanning Electron Microscopy and Energy Dispersive X Ray Spectroscopy - An in vitro study. J Pharm Bioallied Sci.

[167] Kemuriyama S, Aita H, Maida T, Kawamura N, Nezu T, Iijima M (2023). Effect of photofunctionalization on titanium bone-implant integration in ovariectomized rats. Dent Mater J.

[168] Khalap S, Mootha A, Dugal R (2015). Ultraviolet photofunctionalization of Dental Implant surfaces: a review. Jounal Implant Adv Clin Dentistry.

[169] Kim MY, Choi H, Lee JH, Kim JH, Jung HS, Kim JH (2016). UV photofunctionalization effect on bone graft in critical one-wall defect around Implant: a pilot study in Beagle Dogs. Biomed Res Int.

[170] Lee JB, Jo YH, Choi JY, Seol YJ, Lee YM, Ku Y et al. The effect of ultraviolet photofunctionalization on a titanium dental implant with machined surface: an in vitro and in vivo study. Mater (Basel). 2019;12(13). 10.3390/ma12132078 PMC6650865 31261627

[171] Paul V, Mathew TA, Rasheed N, Thomas AS, George N (2023). Photofunctionalization of Dental Implant surfaces - a histomorphometric animal study. J Pharm Bioallied Sci.

[172] Razali M, Ngeow WC, Omar RA, Chai WL. An in-vitro analysis of peri-implant mucosal seal following photofunctionalization of zirconia abutment materials. Biomedicines. 2021;9(1). 10.3390/biomedicines9010078 PMC7830892 33467486

[173] Roy M, Pompella A, Kubacki J, Piosik A, Psiuk B, Klimontko J (2017). Photofunctionalization of dental zirconia oxide: surface modification to improve bio-integration preserving crystal stability. Colloids Surf B Biointerfaces.

[174] Shen JW, Chen Y, Yang GL, Wang XX, He FM, Wang HM. Effects of storage medium and UV photofunctionalization on time-related changes of titanium surface characteristics and biocompatibility. J Biomed Mater Res B Appl Biomater. 2015. 10.1002/jbm.b.33437 25969950

[175] Tuna T, Wein M, Swain M, Fischer J, Att W (2015). Influence of ultraviolet photofunctionalization on the surface characteristics of zirconia-based dental implant materials. Dent Mater.

[176] Suzumura T, Matsuura T, Komatsu K, Ogawa T. A novel high-energy vacuum ultraviolet light photofunctionalization approach for decomposing organic molecules around titanium. Int J Mol Sci. 2023;24(3). 10.3390/ijms24031978 PMC9916712 36768297

[177] Kitajima H, Ogawa T (2016). The Use of Photofunctionalized implants for low or extremely low primary Stability cases. Int J Oral Maxillofac Implants.

[178] Hori N, Iwasa F, Tsukimura N, Sugita Y, Ueno T, Kojima N (2011). Effects of UV photofunctionalization on the nanotopography enhanced initial bioactivity of titanium. Acta Biomater.

[179] Kitajima H, Hirota M, Komatsu K, Isono H, Matsuura T, Mitsudo K (2023). Ultraviolet Light Treatment of Titanium Microfiber Scaffolds enhances osteoblast recruitment and osteoconductivity in a Vertical Bone Augmentation Model: 3D UV photofunctionalization. Cells.

[180] Komatsu K, Matsuura T, Ogawa T. Achieving complete human gingival fibroblast collagen coverage on implant abutments through vacuum ultraviolet (VUV) photofunctionalization. Int J Oral Max Impl. in press. 10.11607/jomi.10957 38657133

[181] Kitajima H, Hirota M, Iwai T, Mitsudo K, Saruta J, Ogawa T (2023). Synergistic Enhancement of Protein Recruitment and Retention via Implant Surface Microtopography and Superhydrophilicity in a computational Fluid Dynamics Model. Int J Mol Sci.

[182] Choi B, Lee YC, Oh KC, Lee JH (2021). Effects of photofunctionalization on early osseointegration of titanium dental implants in the maxillary posterior region: a randomized double-blinded clinical trial. Int J Implant Dent.

[183] Lang X, Qiao B, Ge Z, Yan J, Zhang Y. Clinical effects of photofunctionalization on implant stability and marginal bone loss: systematic review and meta-analysis. J Clin Med. 2022;11(23). 10.3390/jcm11237042 PMC9739233 36498616

[184] Suzuki S, Kobayashi H, Ogawa T (2013). Implant stability change and osseointegration speed of immediately loaded photofunctionalized implants. Implant Dent.

[185] Hirota M, Ikeda T, Tabuchi M, Nakagawa K, Park W, Ishijima M (2016). Bone generation profiling around Photofunctionalized Titanium Mesh. Int J Oral Maxillofac Implants.

[186] Funato A, Tonotsuka R, Murabe H, Hirota M, Ogawa T (2014). A novel strategy for bone integration and regeneration-photofunctionalization of dental implants and Ti mesh. J Cosmet Dent.

[187] Hirota M, Ikeda T, Tabuchi M, Iwai T, Tohnai I, Ogawa T (2014). Effect of ultraviolet-mediated photofunctionalization for bone formation around medical titanium mesh. J Oral Maxillofac Surg.

[188] Hirota M, Ikeda T, Tabuchi M, Ozawa T, Tohnai I, Ogawa T (2017). Effects of Ultraviolet photofunctionalization on bone augmentation and Integration Capabilities of Titanium Mesh and implants. Int J Oral Maxillofac Implants.

[189] Okubo T, Tsukimura N, Taniyama T, Ishijima M, Nakhaei K, Rezaei NM (2018). Ultraviolet treatment restores bioactivity of titanium mesh plate degraded by contact with medical gloves. J Oral Sci.

[190] Park W, Ishijima M, Hirota M, Soltanzadeh P, Ogawa T (2016). Engineering bone-implant integration with photofunctionalized titanium microfibers. J Biomater Appl.

[191] Roy M, Kubacki J, Psiuk B, Mrozek-Wilczkiewicz A, Malarz K, Corti A (2021). Photofunctionalization effect and biological ageing of PEEK, TiO(2) and ZrO(2) abutments material. Mater Sci Eng C Mater Biol Appl.

[192] Tateshima S, Kaneko N, Yamada M, Duckwiler G, Vinuela F, Ogawa T (2018). Increased affinity of endothelial cells to NiTi using ultraviolet irradiation: an in vitro study. J Biomed Mater Res A.

[193] Hirota M, Hori N, Sugita Y, Ikeda T, Park W, Saruta J et al. A novel cell delivery system exploiting synergy between fresh titanium and fibronectin. Cells. 2022;11(14). 10.3390/cells11142158 PMC9317518 35883601

[194] Buser D, Broggini N, Wieland M, Schenk RK, Denzer AJ, Cochran DL (2004). Enhanced bone apposition to a chemically modified SLA titanium surface. J Dent Res.

[195] Schwarz F, Wieland M, Schwartz Z, Zhao G, Rupp F, Geis-Gerstorfer J et al. Review: potential of chemically modified hydrophilic surface characteristics to support tissue integration of titanium dental implants. J Biomed Mater Res B Appl Biomater. 2008. 10.1002/jbm.b.31233 18837448

[196] Suzuki T, Kubo K, Hori N, Yamada M, Kojima N, Sugita Y (2010). Nonvolatile buffer coating of titanium to prevent its biological aging and for drug delivery. Biomaterials.

